# Whole-genome resequencing and transcriptome analyses of four generation mutants to reveal spur-type and skin-color related genes in apple (*Malus domestica* Borkh. Cv. Red delicious)

**DOI:** 10.1186/s12870-023-04631-y

**Published:** 2023-11-30

**Authors:** Jiaxuan Ren, Wenfang Li, Zhigang Guo, Zonghuan Ma, Dongshi Wan, Shixiong Lu, Lili Guo, Huimin Gou, Baihong Chen, Juan Mao

**Affiliations:** 1 College of Horticulture, Gansu Agricultural University, Lanzhou, 730070 PR China; 2 Tianshui Normal University, Tianshui, 741001 PR China; 3 College of Ecology, Lanzhou University, Lanzhou, 730000 PR China

**Keywords:** Apple, Bud sport, Variation analysis, Phytohormone, Anthocyanidins

## Abstract

**Background:**

Bud sport is a kind of somatic mutation that usually occurred in apple. ‘Red Delicious’ is considered to be a special plant material of bud sport, whereas the genetic basis of plant mutants is still unknown. In this study, we used whole-genome resequencing and transcriptome sequencing to identify genes related to spur-type and skin-color in the ‘Red Delicious’ (G0) and its four generation mutants including ‘Starking Red’ (G1), ‘Starkrimson’ (G2), ‘Campbell Redchief’ (G3) and ‘Vallee Spur’ (G4).

**Results:**

The number of single nucleotide polymorphisms (SNPs), insertions and deletions (InDels) and structural variations (SVs) were decreased in four generation mutants compared to G0, and the number of unique SNPs and InDels were over 9-fold and 4-fold higher in G1 versus (vs.) G2 and G2 vs. G3, respectively. Chromosomes 2, 5, 11 and 15 carried the most SNPs, InDels and SVs, while chromosomes 1 and 6 carried the least. Meanwhile, we identified 4,356 variation genes by whole-genome resequencing and transcriptome, and obtained 13 and 16 differentially expressed genes (DEGs) related to spur-type and skin-color by gene expression levels. Among them, *DELLA* and *4CL7* were the potential genes that regulate the difference of spur-type and skin-color characters, respectively.

**Conclusions:**

Our study identified potential genes associated with spur-type and skin-color differences in ‘Red Delicious’ and its four generation mutants, which provides a theoretical foundation for the mechanism of the apple bud sport.

**Supplementary Information:**

The online version contains supplementary material available at 10.1186/s12870-023-04631-y.

## Background

Plant tissues and organs are prone to somatic mutations due to external conditions during frequent cell divisions. In this process, bud sport is a somatic mutation that usually occurs in bud meristem cells of plants. Previous research has shown that bud sport is an important source of information for breeding new cultivars or strains that are superior to the parent [1]. In contrast with animal systems, some somatic mutations are highly likely to be transmitted the next generation in higher plants. This is because the meristematic tissues can produce all tissues, including reproductive tissues, and as a result of unlimited growth and totipotency of the plant cells during development [2, 3]. Somatic mutation is a single mutation caused by the change of genetic material in the nucleus. Studies have found that it always occurs in meristems of the developing shoot, and directly or indirectly influences the genes involved in related biosynthesis [4]. In most cases, the sports differ from the mother plant strain in monogenic character, while two or several genes or gene complexes are involved in special cases [5]. Previous studies have confirmed somatic mutations usually cause changes of branching habit, fruit color, shape and maturity [1, 6, 7].

‘Red Delicious’ (*Malus domestica* Brokh. Cv. Red delicious) is typical bud sport variety. It was first discovered growing as a seedling tree in an orchard in the Iowa, USA in 1872. The sport variety of ‘Red Delicious’ contains four generation mutants (Supplementary Fig. 1). ‘Starking Red’ is a typical representative of the first generation derived from ‘Red Delicious’ via somatic mutant or sport, including ‘Richared’ (1915), ‘Red Prince’ (1955) and other more than 30 mutants mostly are coloration sports. The second generation contains ‘Starkrimson’ (1953), ‘Redking’ (1953), and other more than 60 mutants. They came from bud sports of ‘Starking Red’ and mostly are the spur-types. The third generation contains ‘Redspar’ (1954), ‘Campbell Redchief’ (1967), ‘Red Chief’ (1974), and other more than 20 mutants. ‘Vallee Spur’ (1989) is a bud sport variety derived from ‘Campbell Redchief’ and is considered the fourth generation mutant of ‘Red Delicious’, which contains more than 10 mutants up to now [8]. Obviously, the mutant of ‘Red Delicious’ is usually selected according to the spur-type and dark-red fruit color. Therefore, it is great significance to work on the somatic mutation of ‘Red Delicious’ and its four generation mutants.

Apple spur-type bud mutation is a genetic chimera caused by mutations in the genetic material of somatic cells [3]. Phytohormones interaction leads to phenotypic differences between dwarfing mutants and wild-type plants [9]. Many studies have shown that the phenomenon of shorter internode is closely related to the gibberellin (GA) and brassinosteroid (BR) in plants. Schomburg et al. [10] found that overexpression of GA2-oxidase (*GA2ox*), as a rate-limiting enzyme of GA metabolic pathway, leads to shorter internode in rice. Meanwhile, it has been proposed that *OsBRI1* gene was involved in internode elongation of rice by inducing stem meristem formation and longitudinal elongation of internode cells [11]. So far, the regulation of phytohormone on growth and development has been extensively studied in various plants. Skin-color is an important factor for determining the commodity value of fruit. Anthocyanins are usually synthesized on the cytoplasmic surface and then modified by methylation, glycosylation, hydroxylation and acetylation to form stable anthocyanins in the endoplasmic membrane [12]. Finally, anthocyanin glycosides are accumulated in the vesicles with the assistance of transport proteins and transport vesicles to give flowers and fruits a variety of colors in plants [13, 14]. Anthocyanin synthesis is regulated by multiple enzymes such as phenylalanine aminolyase (PAL), 4-coumarate CoA ligase (4CL), chalcone synthase (CHS), chalcone isomerase (CHI), flavanone-3-hydroxylase (F3H), flavanone-3’-hydroxylase (F3’H), flavonoid-3’,5’-hydroxylase (F3’5’H), dihydroflavanol 4-reductase (DFR), anthocyanidin synthase (ANS) and flavanone-3’-hydroxylase (UFGT), and is encoded by genes present in multiple gene families [15]. In contrast, *DFR*, *ANS* and *UFGT* genes were found to be efficiently expressed when the fruit showed bright colors [16, 17]. The accumulation of anthocyanins in fruits is the result of the efficient expression of various key enzymes for their biosynthesis, and the expression of these key enzymes is closely related to the role of regulatory genes. However, the mechanism of many differences between somatic mutant materials and parents remains to be further explored.

Using whole-genome resequencing technology, individual genomes can be quickly screened, variant genes can be found, and variant types can be detected, including single nucleotide polymorphisms (SNPs) sites and insertion and deletion variants (InDels) mutations, so as to achieve functional gene prediction and genetic evolution analysis [18]. At present, whole-genome resequencing technology has been widely used to analyze the differences between different individuals or groups of plant with known genome sequences. For example, Lim et al. [19] used next generation sequencing technology to conduct whole-genome resequencing of two parent varieties to identify major effector quantitative trait locus (QTLs) for shape-related traits in rice plants and to search for candidate genes. Moreover, whole-genome resequencing technique was used to study osmanthus flower color, and the authors identified significant QTL and genomic regions governing genes such as ethylene response transcription factor 2 and Arabidopsis pseudo response regulator 2, which were positively correlated with petal color [20]. In addition, they also found a frameshift with 34 bp deletion in the first coding region of the carotenoid lytic dioxygenase 4 gene [20]. Transcriptome sequencing (RNA-Seq) technology, as a high-throughput sequencing technology, can detect the gene expression of a cell or tissue in a specific state. In addition, the technology can uncover new genes, accurately identify SNPs, and provide comprehensive transcriptional information [21]. Because RNA-Seq has the advantages of short sequencing cycle, high accuracy and no species restriction, it has been widely used. Wei et al. [22] revealed the effects of salt stress on banana leaf by transcriptome sequencing, and the results showed that a total of 3,378 differentially expressed genes (DEGs) were identified in banana leaves. Meanwhile, these DEGs are involved in the process of phenylC biosynthesis, ribosome, starch and sucrose metabolism, amino sugar and plant hormone signal transduction, and may play a role in promoting the growth of banana under salt treatment [22]. Importantly, the combination of whole-genome resequencing and transcriptome analyses can reveal the genetic mechanisms of multiple metabolite directed changes in plant mutation, evolution and natural selection. For example, Zhao et al. [23] combined resequencing and transcriptome analysis of ‘Zhonglin 1’ and ‘RW-1’ walnuts and identified gene encoding anthocyanidin 3-O-glucosyltransferase. The expression pattern was positively correlated with the accumulation of anthocyanins in the skins of different walnuts. In a word, the whole-genome resequencing and transcriptome analyses technology have been widely used in the study of plant variation traits and genes.

In recent years, a large number of studies have confirmed and reported that ‘Red Delicious’ is a kind of special plant material derived from the bud sport mutation [24]. However, so far, there are few studies on the four generation mutants of ‘Red Delicious’, and the variation amplitude and pattern are still unknown. In this study, whole-genome resequencing and transcriptome analysis were used to reveal differences in spur-type and skin-color in ‘Red Delicious’ and its four generation mutants. By analyzing the SNPs, InDels, and expression levels of hormone signal transduction and anthocyanin synthesis genes, DEGs with structural differences between ‘Red Delicious’ and its four generation mutants were identified, and further screened for genes related to the regulation of spur-type and skin-color. This provides a research direction and molecular basis for further studies on the mechanism of bud variation and breeding in apple.

## Results

### Bud sport selection leads to altered phenotypes among ‘Red Delicious’ and its four generation mutants

**Fig. 1:**
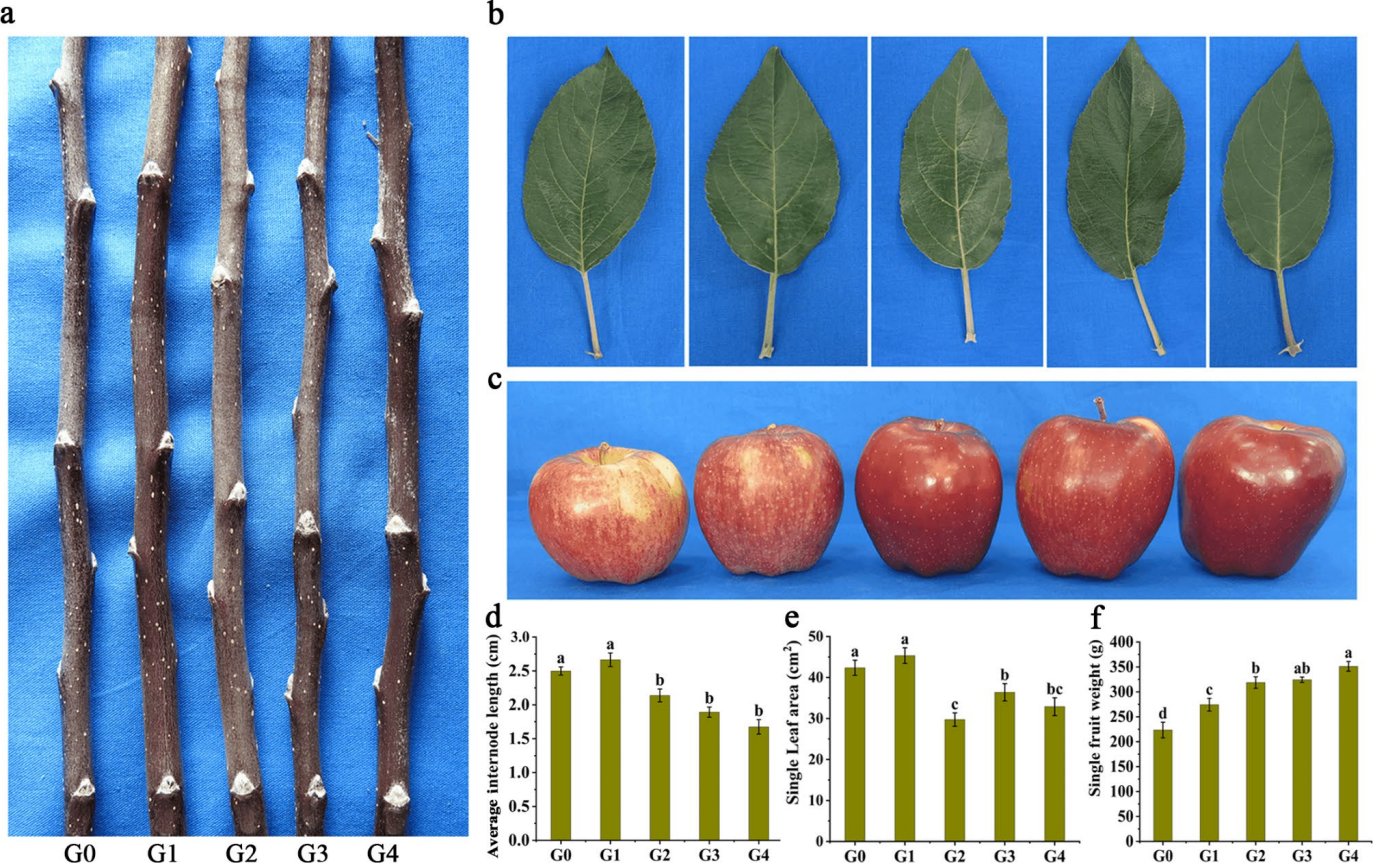
Apparent difference of branches (**a**), leaves (**b**), fruits (**c**), average internode length (**d**), single leaf area (**e**) and single fruit weight (**f**) in ‘Red Delicious’ and its four generation mutants. The figures from left to right are ‘Red Delicious’ (G0), ‘Starking red’ (G1), ‘Starkrimson’ (G2), ‘Campbell Redchief’ (G3), ‘Vallee spur’ (G4). Fifteen trees of each generation were chose to collect branches, leaves and fruit. A replicate contains five fruit trees, and set three independent biological replicates. The values of three independent experiments were expressed as the average ± SE. Different small letters within the indicate significant differences at *p* < 0.05

In order to identify the differences between ‘Red Delicious’ and its four generation mutants, we compared their branches, leaves and fruits. As is shown in Fig. 1a, b, compared to G0, the average internode length and single leaf area were significantly decreased in G2, G3 and G4, but there was no significant difference between G0 and G1. Additionally, the color of fruit skin gradually deepened as the number of mutant generations increased, and the fruit appearance of G0 and G1 were striped red, while G2, G3 and G4 were full red (Fig. 1c). Meanwhile, the single fruit weight was significantly increased with the increase of mutant generations. Specifically, compared with G0 (223.47 g), the single fruit weight significantly increased by 22.73%, 42.60%, 45.05% and 57.13% with the increase of mutant generation, respectively (Fig. 1f). These results suggested that the bud sport selection among ‘Red Delicious’ and its four generation mutants maybe the main cause of the branches, leaves and fruits alteration phenotype.

### Whole-genome variation among ‘Red Delicious’ and its four generation mutants

**Fig. 2:**
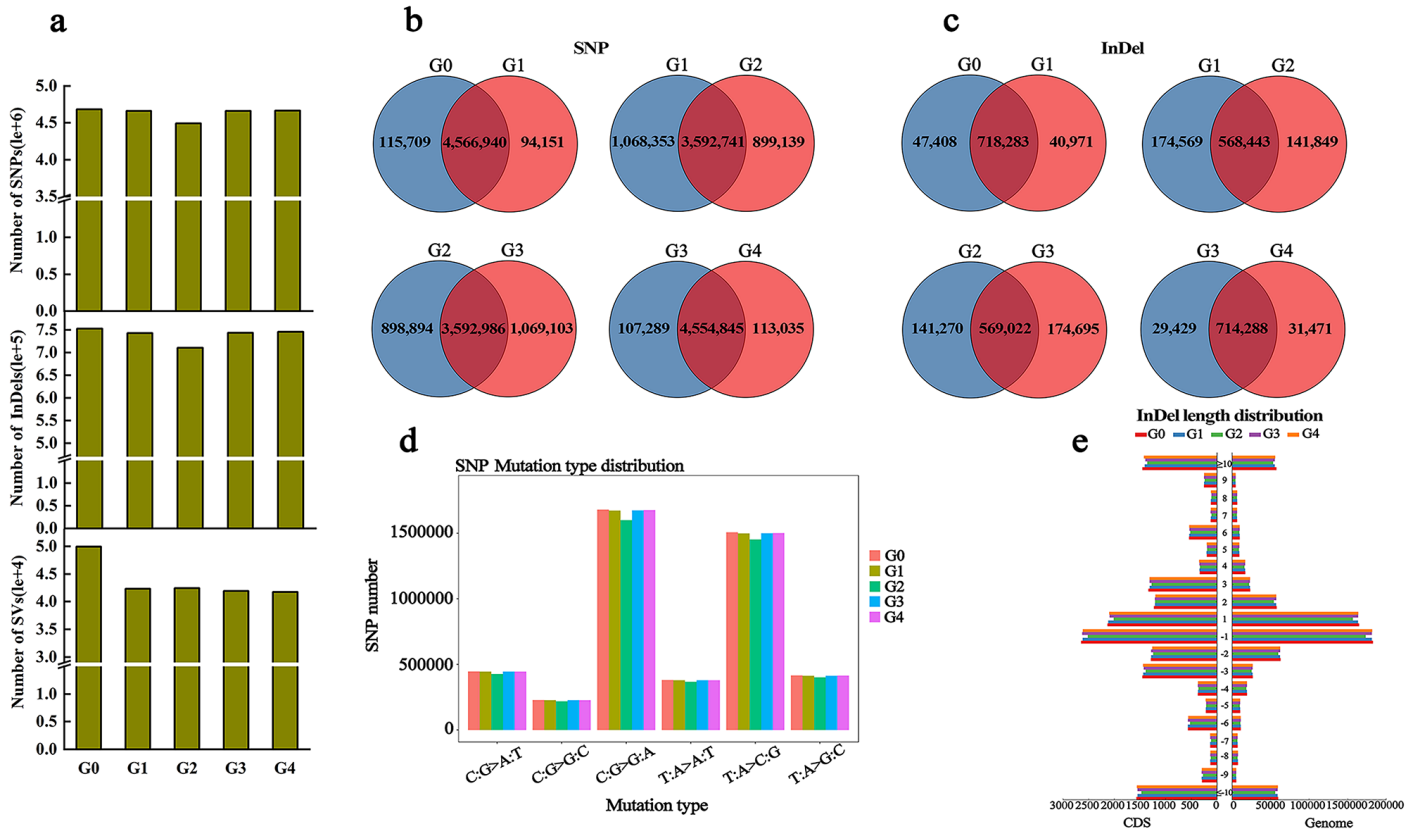
Variation statistic and distribution. G0, G1, G2, G3 and G4 represent ‘Red Delicious’, ‘Starking red’, ‘Starkrimson’, ‘Campbell Redchief’ and ‘Vallee spur’, respectively. **a** Statistics on the various numbers of SNPs, InDels and SVs in five samples. **b** Venn diagram of numbers of shared and specific SNPs between each generation. **c** Venn diagram of numbers of shared and specific InDels between each generation. **d** Distribution of SNP mutation type in five samples. The X-axis indicates the type of SNP mutation, and the Y-axis indicates the number of SNPs. **e** InDels length distribution of CDS and genome in five samples

To further explore the sources responsible for the differences in these mutants, Illumina HiSeq 2,000 was used to sequence the ‘Red Delicious’ and its four generation mutants, and all data were quality-controlled to get clean data. The 213 Gb raw data from sequencing was filtered to obtain 212.702 Gb clean data, and the raw reads of each sample ranging from 32.927 Gb to 53.026 Gb. The GC content, ranging from 38.18–38.29% was moderate, and the sequencing results of Q20 ≥ 96.76% and Q30 ≥ 92.27% were considered high-quality (Supplementary Table 1). Furthermore, the sequencing reads of all samples were aligned to the apple reference genome GDDH13 v1.1, the mapping rate ranging from 96.90–97.72%. The average genome (excluding N) coverage depth of the five samples ranged from 46.00× to 74.00×, 1× coverage ratio was over 96.39% (Table 1; Supplementary Table 2). Consequently, SNP calling and variant analysis identified 4,491,880-4,682,649 SNPs, 710,292–752,869 InDels and 41,760 − 49,921 SVs in individual samples. Furthermore, the number of SNPs, InDels and SVs were decreased in the four generation mutants relative to G0 (Table 1; Fig. 2a).

**Table 1:** Summary of sequencing results for ‘Red Delicious’ and its four generation mutants

Sample	Common name	Mapped reads	Average depth (×)	GC Content (%)	SNPs	InDels	SVs
G0	Red Delicious	342,088,246 (96.90%)	74.00	38.18	4,682,649	752,869	49,921
G1	Starking Red	217,331,395 (97.40%)	46.00	38.23	4,661,094	743,012	42,305
G2	Starkrimson	228,859,957 (97.67%)	48.00	38.22	4,491,880	710,292	42,453
G3	Campbell Redchief	214,202,597 (97.72%)	46.00	38.27	4,662,134	743,717	41,914
G4	Vallee spur	227,272,380 (97.60%)	49.00	38.29	4,667,880	745,759	41,760

### Identification and assessment of SNPs, InDels and SVs among ‘Red Delicious’ and its four generation mutants

Based on the above evaluation of sequencing data, the statistics of unique SNPs and InDels of each mutant versus (vs.) to G0 were shown in Supplementary Fig. 2. Compared with G0 vs. G1, G0 vs. G3 and G0 vs. G4, the number of unique SNPs and InDels were over 9-fold and 3-fold higher in G0 vs. G2, respectively. Similarly, the number of unique SNPs and InDels were over 9-fold and 4-fold higher in G1 vs. G2 and G2 vs. G3, respectively (Fig. 2b, c). The number of transition (Ti) and transversion (Tv) SNPs ranged from 3,062,229 to 3,196,395 and 1,429,651 to 1,486,254, respectively. The Ti/Tv ratio is between 2.142 and 2.151, and the heterozygous rate of SNPs ranging from 2.342‰ to 2.444‰ (Supplementary Table 3). Additionally, according to the total number of identified InDels, 344,710 − 364,581 insertions (INS) and 381,182–401,110 deletions (DEL) were detected in the five generations (Supplementary Table 4). The distribution of SNP mutation types and CDS InDels length were shown in Fig. 2d, e. Type C: G change to G: A and type T: A change to C: G constituted the majority of the SNPs, and at 1 and − 1 bp InDels length reaches the maximum in CDS and genome.

**Fig. 3:**
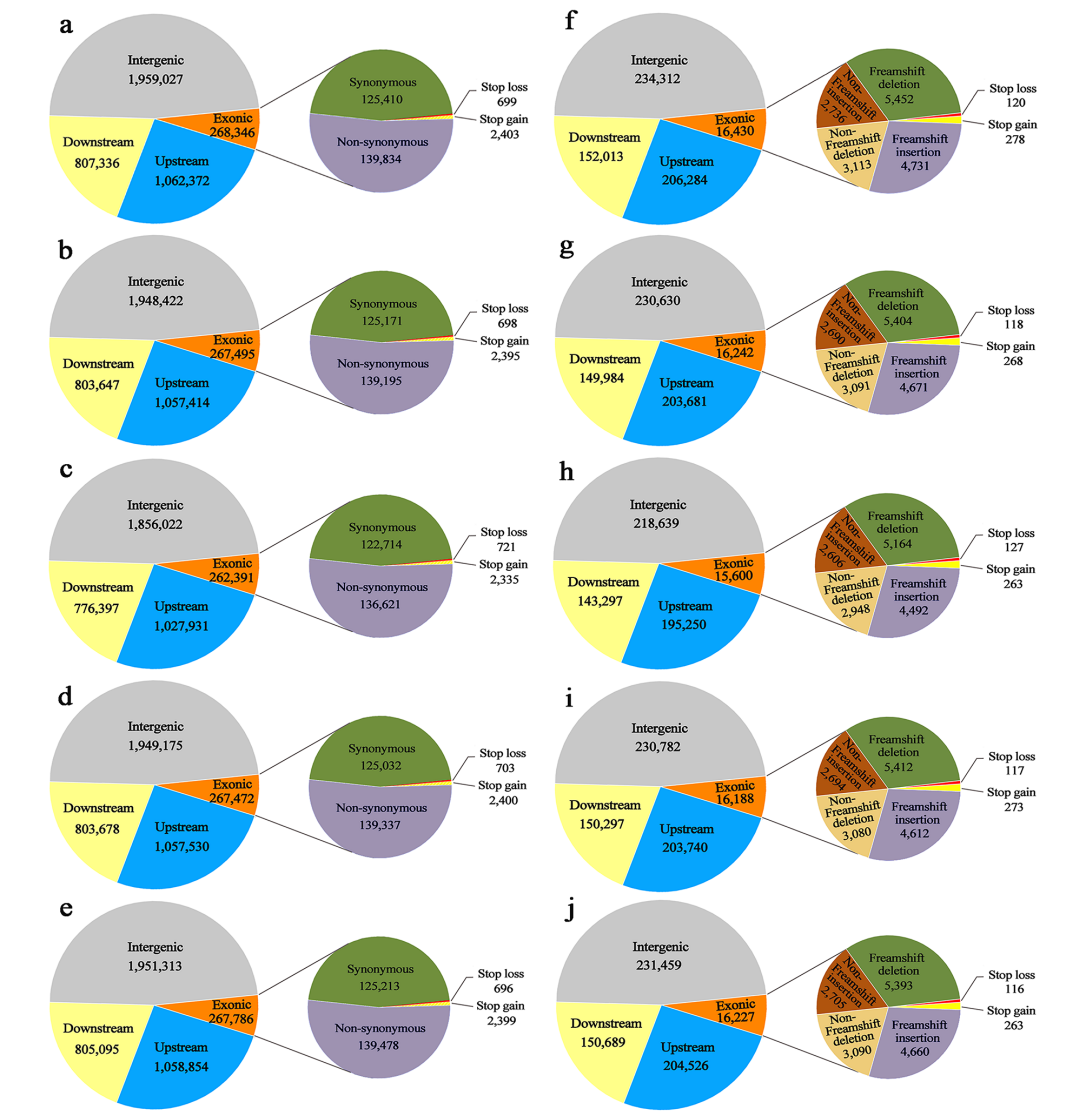
Regional analysis of gene variation. G0, G1, G2, G3 and G4 represent ‘Red Delicious’, ‘Starking red’, ‘Starkrimson’, ‘Campbell Redchief’ and ‘Vallee spur’, respectively. Different colored regions show the SNPs distribution of intergenic region, upstream, downstream and exon in G0 (**a**), G1 (**b**), G2 (**c**), G3 (**d**) and G4 (**e**). Among them, the number of synonymous, non-synonymous, stop gain, and stop loss SNPs detected within the exon region also were shown. Different colored regions show the InDels distribution of intergenic region, upstream, downstream and exon in G0 (**f**), G1 (**g**), G2 (**h**), G3 (**i**) and G4 (**j**). Among them, the number of frameshift mutation, non-frameshift mutation, stop gain, and stop loss InDels detected within the exon also were shown

Among the five generations, approximately 47% SNPs and 38% InDels were identified in intergenic regions, and about 46% SNPs and 59% InDels in the upstream and downstream regulatory regions were found (Fig. 3). Moreover, only approximately 7% SNPs and 3% InDels were identified in the exon region, and about 52% of non-synonymous SNPs and 62% of InDels were identified in the five generations (Fig. 3). Meanwhile, the SVs, with less than two supported paired-end reads, were 41,760 − 49,921 compared to apple reference genome GDDH13 v1.1 (Supplementary Table 5). There were 2,250-3,661, 21,362 − 24,900, 962-1,173, 5,505-7,569, and 10,357 − 12,414 of INS, DEL, inversions (INV), intra-chromosome transfers (ITX) and inter-chromosome transfers (CTX) in individual samples, respectively (Supplementary Table 5).

### The chromosomes bias distribution of the SNPs, InDels and SVs

**Fig. 4:**
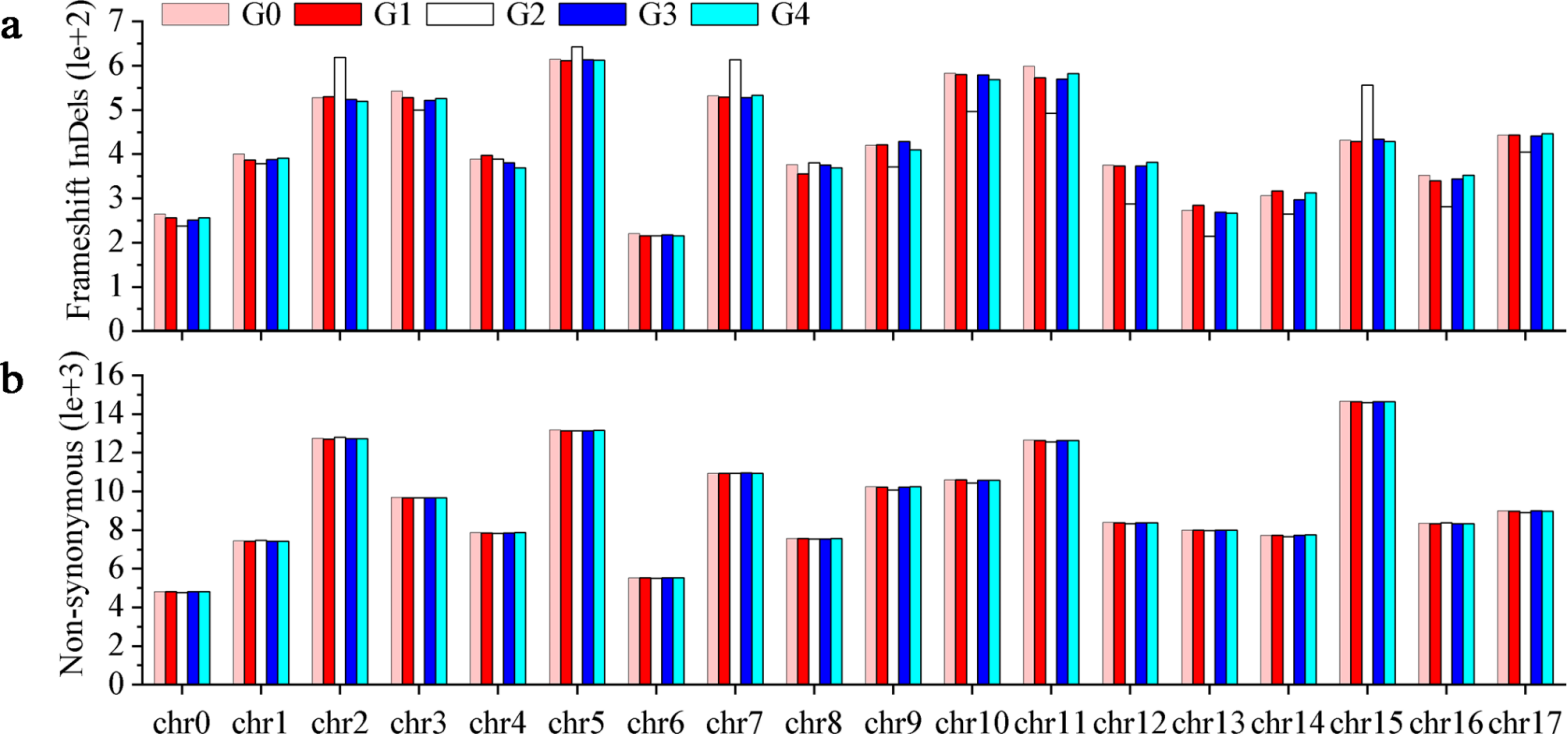
Distribution of detected mutations on 18 chromosomes of the five samples. G0, G1, G2, G3 and G4 represent ‘Red Delicious’, ‘Starking red’, ‘Starkrimson’, ‘Campbell Redchief’ and ‘Vallee spur’, respectively. **a** Number and distribution of InDels. The X-axis indicates chromosome 0 to chromosome 17, and the Y-axis indicates the number of InDels. **b** Number and distribution of non-synonymous SNPs. The X-axis indicates chromosome 0 to chromosome 17, and the Y-axis indicates the number of non-synonymous

Analysis of sequencing results showed that the numbers of non-synonymous SNPs and InDels varied across 18 chromosomes among the five generations (Fig. 4a, b; Table 2). Specifically, chromosome 15 with highest non-synonymous SNPs, followed by chromosomes 5, 2 and 11. In G0, G1, G3 and G4, the maximum number of InDels was detected in chromosome 5, followed by 10 and 11, while chromosome 5, followed by 2, and 7 in G2. For the five generations, the minimum number of non-synonymous SNPs and InDels were detected in chromosome 1, followed by 6 (Fig. 4a, b; Table 2). Moreover, the distribution of SVs on each chromosome also showed higher variability. Chromosomes 10, 11 and 15 had the most SVs (more than 3,352 unique SVs per chromosome), while chromosome 8 carried only a small number of SVs (less than 1,670 unique SVs). The proportion of INV was 1.04-5.15%, followed by INS was 4.35-9.52%, ITX was 9.28-20.66%, CTX was 0.00-43.29%, and DEL was 27.02–77.19% in the five generations (Supplementary Table 6).

**Table 2:** Number and distribution of non-synonymous SNPs and frameshift InDels detected on the apple chromosomes in ‘Red Delicious’ and its four generation mutants

Chr	G0				G1				G2				G3				G4			
	Ns	Frameshift			Ns	Frameshift			Ns	Frameshift			Ns	Frameshift			Ns	Frameshift		
		Del	Ins	Sub		Del	Ins	Sub		Del	Ins	Sub		Del	Ins	Sub		Del	Ins	Sub
Chr0	4,813	73	130	1	4,798	75	128	1	4,764	64	129	2	4,806	73	129	0	4,808	75	182	1
Chr1	7,435	274	231	5	7,420	267	215	5	7,468	252	214	4	7,412	264	222	4	7,421	263	218	6
Chr2	12,753	250	202	4	12,707	246	192	2	12,791	252	172	3	12,721	246	189	4	12,725	247	183	5
Chr3	9,685	151	102	3	9,655	154	108	4	9,659	139	157	4	9,670	158	108	2	9,675	147	118	3
Chr4	7,863	21	26	4	7,859	22	28	0	7,823	25	27	0	7,852	22	25	0	7,866	23	26	0
Chr5	13,170	231	180	0	13,134	226	179	2	13,135	249	196	4	13,130	223	175	3	13,146	224	183	4
Chr6	5,525	142	122	5	5,512	146	117	3	5,507	140	110	1	5,521	141	117	5	5,520	144	115	4
Chr7	10,940	208	148	1	10,939	202	148	2	10,938	217	145	2	10,944	206	140	2	10,939	205	140	3
Chr8	7,565	202	161	0	7,554	187	157	1	7,545	174	131	2	7,551	202	146	2	7,570	193	159	2
Chr9	10,228	260	193	2	10,224	260	192	2	10,080	252	178	1	10,219	260	197	2	10,228	263	197	5
Chr10	10,589	221	180	3	10,587	216	171	3	10,436	230	170	2	10,582	214	214	1	10,583	214	214	4
Chr11	12,650	271	187	3	12,631	249	180	4	12,544	268	181	2	12,622	254	183	3	12,638	253	182	3
Chr12	8,389	223	165	4	8,384	221	166	2	8,320	210	165	1	8,387	226	155	3	8,382	221	165	6
Chr13	7,997	218	140	4	7,994	217	139	8	7,962	206	130	3	7,991	207	139	9	7,993	215	142	6
Chr14	7,743	185	152	5	7,743	179	150	1	7,657	166	141	1	7,742	171	156	2	7,744	193	151	1
Chr15	14,658	319	248	5	14,621	313	237	6	14,593	365	273	7	14,638	318	239	6	14,634	315	242	7
Chr16	8,343	153	108	2	8,336	152	104	2	8,375	142	96	1	8,334	151	105	1	8,334	154	108	5
Chr17	9,001	189	107	1	8,977	187	109	3	8,900	183	106	2	8,993	193	108	1	8,983	185	107	2

Note: G0, G1, G2, G3 and G4 represent ‘Red Delicious’, ‘Starking red’, ‘Starkrimson’, ‘Campbell Redchief’ and ‘Vallee spur’, respectively. Chr, Chromosome; Ns, Non-synonymous; Del, Deletion; Ins, Insertion; Sub, Substitution

### Gene annotation identified potential genes for spur-type and skin-color mutations

**Fig. 5:**
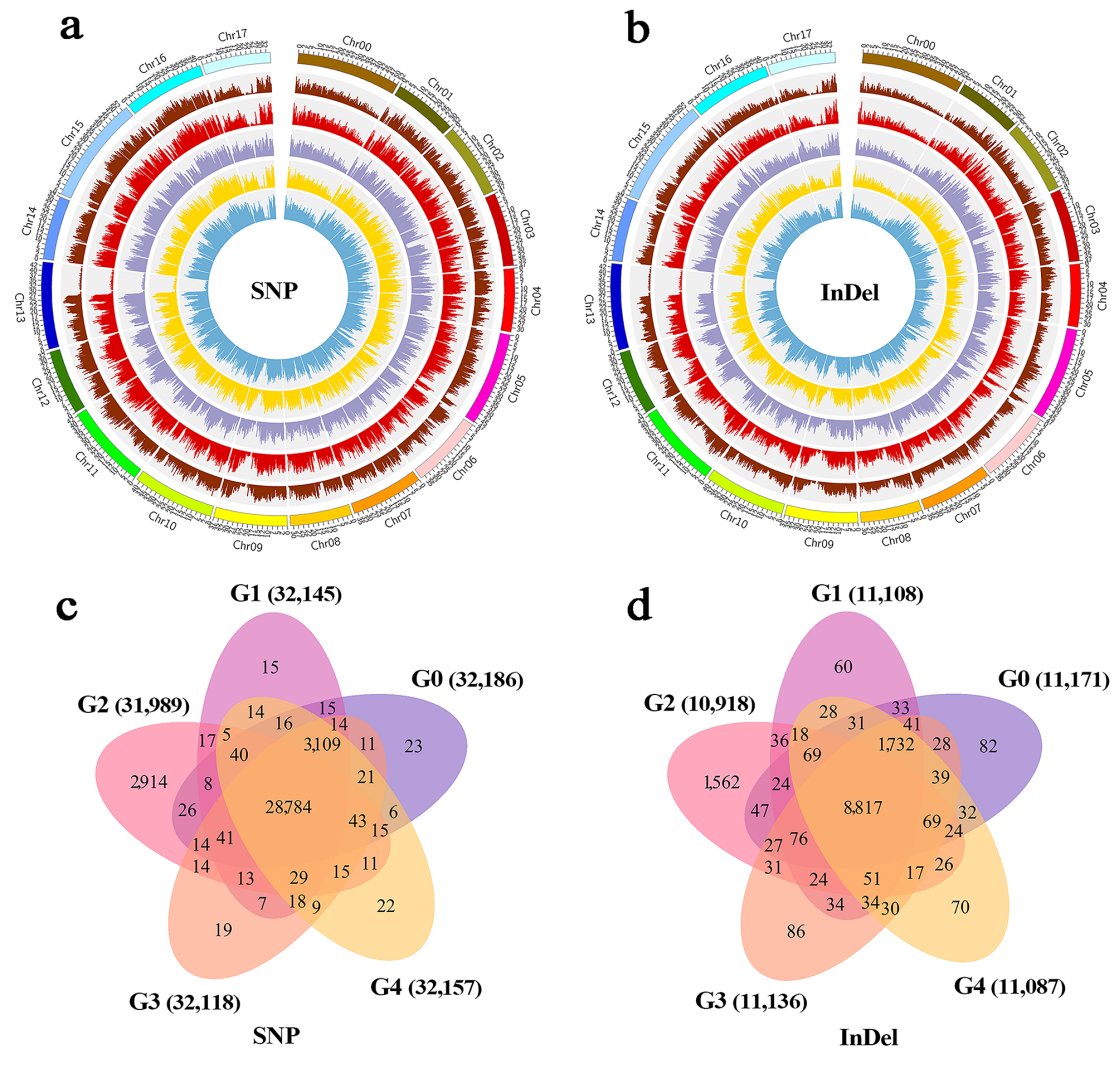
Genetic structure polymorphism density map and gene statistics of ‘Red Delicious’ and its four generation mutants. G0, G1, G2, G3 and G4 represent ‘Red Delicious’, ‘Starking red’, ‘Starkrimson’, ‘Campbell Redchief’ and ‘Vallee spur’, respectively. **a** Density maps of SNP genetic structure polymorphism. **b** Density maps of InDel genetic structure polymorphism. From the inside to the outside, the map shows sample site information of G0, G1, G2, G3 and G4 and chromosome coordinates (labeled with serial numbers), respectively. **c** Venn diagram of the number of shared and specific non-synonymous SNP genes between five samples. **d** Venn diagram of the number of shared and specific frameshift InDel genes between five samples

Mutations occurring in the CDS region may cause changes in gene function, and looking for non-synonymous SNPs and InDels in the CDS region helps to understand the source of functional variation between ‘Red Delicious’ and its four generation mutants. The overall mutation frequency of SNPs and InDels in G0 was higher than that of its four generation mutants, and chromosome 15 of G0, G1, G2, G3 and G4 had a higher density of SNPs and InDels (Fig. 5a, b). Meanwhile, the coverage depth of each chromosomal locus is more evenly distributed across the chromosome, indicating better sequencing randomness (Supplementary Fig. 3). A total of 32,186, 32,145, 31,989, 32,118 and 32,157 genes containing non-synonymous SNPs were detected in G0, G1, G2, G3 and G4, respectively. Meanwhile, 11,171, 11,108, 10,918, 11,136 and 11,087 genes containing InDels were detected from G0, G1, G2, G3 and G4, respectively. Of these, 28,784 and 8,817 common genes were found to contain non-synonymous SNPs and InDels in the five samples, respectively (Fig. 5c, d). In addition, the largest number of non-synonymous SNPs and InDels were identified in G2 vs. G3 (Supplementary Table 7).

We looked for genes that differed between the sample and the reference genome, and functionally annotated the mutated genes at the DNA level. The annotation system of Gene Ontology (GO) is divided into three broad classes: biological processes, molecular function and cellular components. Genes derived from cellular components, molecular functions, and biological processes can usually be classified into 12, 12 and 18 groups, respectively. Among them, cell part (378), catalytic activity (626) and metabolic process (765) accounted for the largest share in cellular component, molecular function and biological process, respectively (Supplementary Fig. 4a). Classification of the Kyoto Encyclopedia of Genes and Genomes (KEGG) pathway for the mutant genes showed that it was mainly associated with ribosome (71), starch and sucrose metabolism (65), carbon metabolism (52), biosynthesis of amino acids (51) and phenylpropanoid biosynthesis (51) (Supplementary Fig. 4b).

### RNA-Seq and identification of DEGs

Our previous studies found that there was a significant difference in fruit skin color between ‘Red Delicious’ and its four generation mutants in the S2 period, and IAA concentration also reached a peak at this period, so we chose the S2 period for RNA-Seq [8]. The results showed that each sample in G0-G4 mapped 83.95% of the reads on average. The results of differential expression analysis showed that a total of 3,485 DEGs were obtained in ‘Red Delicious’ and its four generation mutants, of which 1,456 were up-regulated and 2,029 were down-regulated genes. Among them, the number of both up- and down-regulated DEGs increased significantly from G1 to G4.

### Gene expression analysis of the related SNPs and InDels variation with plant characteristic of ‘Red Delicious’ and its four generation mutants

**Fig. 6:**
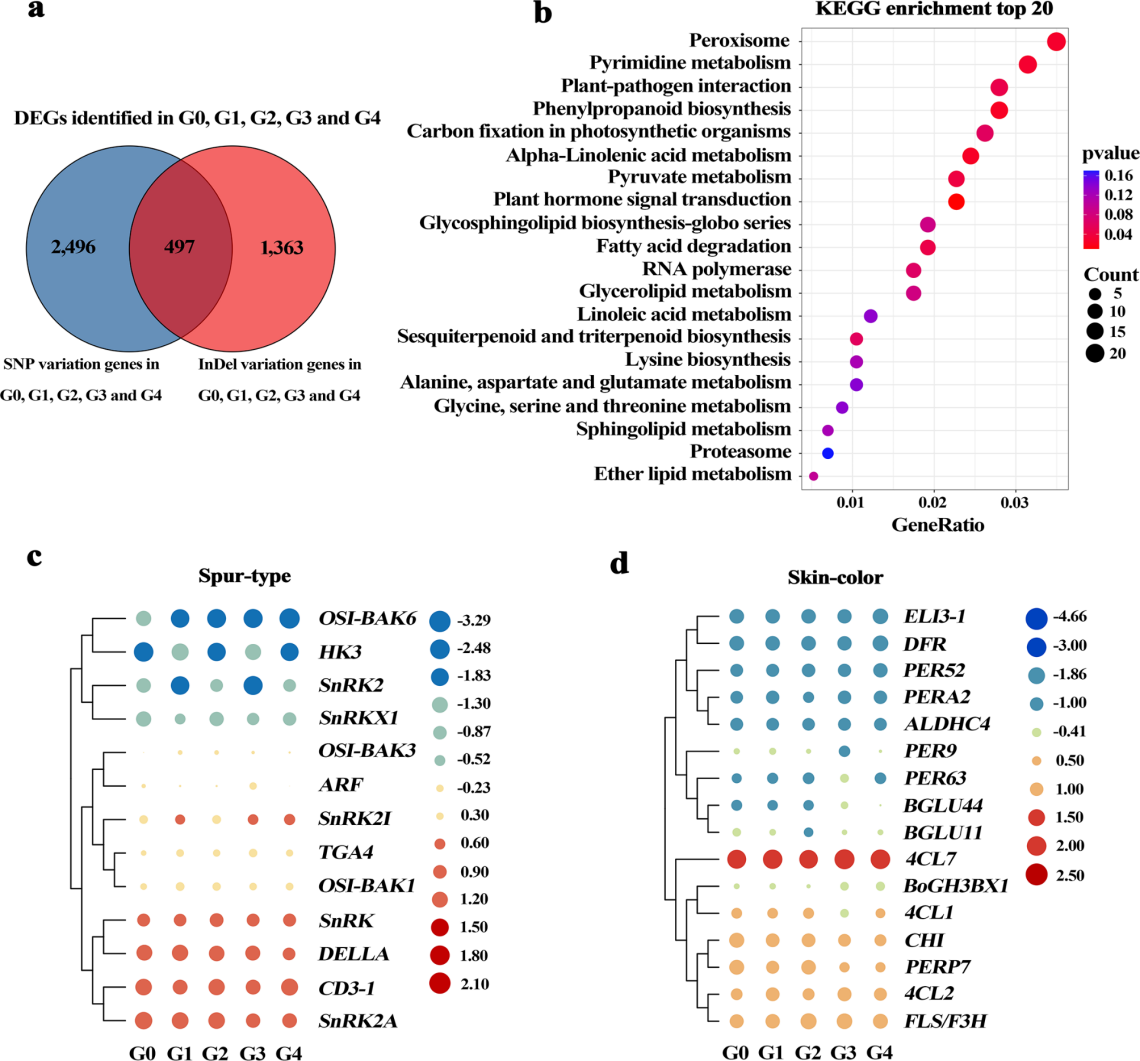
Analysis of structural variants DEGs associated with spur-type and skin-color. G0, G1, G2, G3 and G4 represent ‘Red Delicious’, ‘Starking red’, ‘Starkrimson’, ‘Campbell Redchief’ and ‘Vallee spur’, respectively. **a** Specific SNP and InDel variation genes were shown in blue and red in five samples, respectively. **b** KEGG enrichment analysis of 4,356 genes. The X-axis indicates GeneRatio. The size of the bubble represents the number of genes, and the color of the bubble from purple to red indicates the smaller pvalue and the greater of significance. The KEGG pathway map was sourced from KEGG Mapper (https://www.kegg.jp/kegg/mapper/), and we have obtained written permission to use and adapt it [25–27]. **c** Expression levels of 13 genes related to spur-type. **d** Expression levels of 16 genes related to skin-color. Gene expression levels were calculated by using log_2_ FPKM values, and red and blue represents up-regulated and down-regulated genes, respectively

We further identified DEGs with structural differences between G0, G1, G2, G3 and G4 by combining whole-genome resequencing and transcriptome. A total of 2,993 SNPs and 1,860 InDels variation genes were identified among G0, G1, G2, G3 and G4 compared to the apple reference genome GDDH13 v1.1, and 497 genes were shared (Fig. 6a). Therefore, total 4,356 variation genes with SNPs and InDels among G0, G1, G2, G3 and G4 were obtained. After that, KEGG enrichment analysis was performed on 4,356 genes. As shown in Fig. 6b, DEGs was significantly enriched in plant hormone signal transduction and phenylpropanoid biosynthesis pathways. Therefore, we identified 13 and 16 DEGs enriched in plant hormone signaling and phenylpropanoid biosynthesis pathways as spur-type and skin-color difference-related genes, respectively (Supplementary Table 8). We analyzed gene expression levels at log_2_ Fragments Per Kilobase of transcript per Million mapped reads (FPKM) values for G0, G1, G2, G3 and G4 samples. The results showed that expression levels of spur-type genes *SnRK2A* (MD15G1428500), *CD3-1* (MD05G1087300), *DELLA* (MD16G1023300) and *SnRK* (MD10G1134600) were significantly up-regulated (Fig. 6c).

Cluster analysis of 16 genes related to skin-color showed that *4CL7* (MD11G1145900), *FLS/F3H* (MD15G1353800), *4CL2* (MD07G1309000), *PERP7* (MD01G1162100) and *CHI* (MD01G1118300) were significantly up-regulated, and the expression level of *4CL7* was the highest (Fig. 6d). To determine the expression levels of DEGs, four spur-type and five skin-color related genes were selected for qRT-PCR analysis, respectively. Among them, *DELLA* gene expression decreased gradually from G1 to G4, and *4CL7* gene expression decreased first and then increased from G0 to G4. The results of qRT-PCR analysis shown that the expression patterns of all these genes were consistent with the RNA-Seq data (Supplementary Fig. 5). Therefore, the results of gene expression analysis indicated that *DELLA* and *4CL7* were potential genes related to spur-type and skin-color, respectively.

### Variation analysis of *DELLA* and *4CL7* in ‘Red Delicious’ and its four generation mutants

**Fig. 7:**
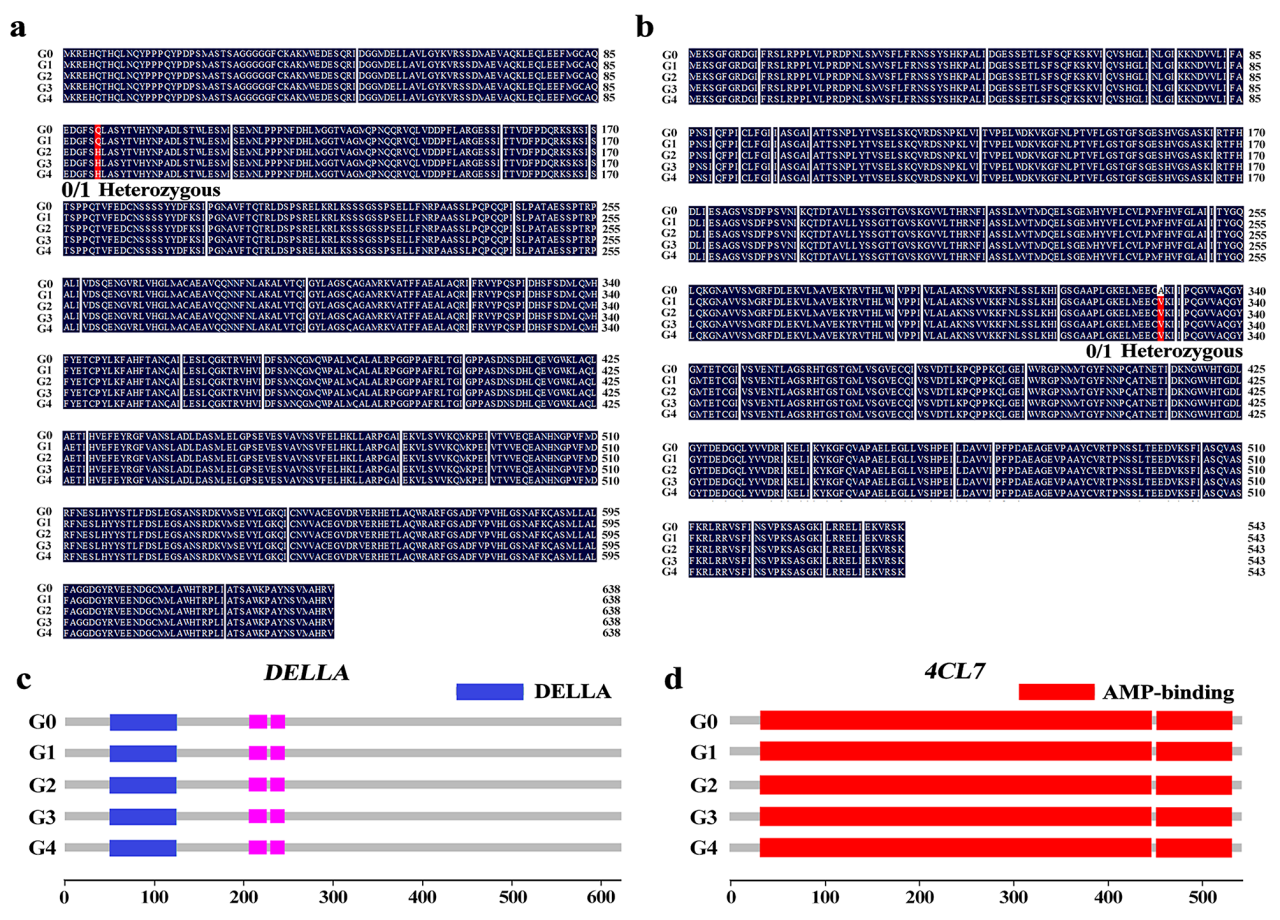
Sequence alignment and domain analysis of *DELLA* and *4CL7* in ‘Red Delicious’ and its four generation mutants. G0, G1, G2, G3 and G4 represent ‘Red Delicious’, ‘Starking red’, ‘Starkrimson’, ‘Campbell Redchief’ and ‘Vallee spur’, respectively. **a** DELLA protein multiple sequence alignments. **b** 4CL7 protein multiple sequence alignments. Blue represents the same amino acids, and red represents different amino acids. **c** Prediction of *DELLA* domain in G0-G4. **d** Prediction of *4CL7* domain in G0-G4. The blue square is the DELLA domain, and the red square is the AMP-bing domain

In order to confirm the whole-genome resequencing results, we verified the non-synonymous SNP loci and InDel of *DELLA* gene. The results showed that frameshift insertion occurred at 1,699,443 bp in G0 vs. G2, G1 vs. G2 and G2 vs. G3, and base G/T substitution occurred at 1,699,278 bp of the *DELLA* CDS region in G2-G4 (Supplementary Tables 8, 10). Meanwhile, the frameshift deletion occurred at 13,686,893 bp in G0 vs. G1, G0 vs. G2 and G2 vs. G3, and base C/T substitution occurred at 13,694,480 bp of the *4CL7* CDS region in G1-G4 (Supplementary Tables 8, 10). We isolated the CDS sequences of *DELLA* and *4CL7* from ‘Red Delicious’ and its four generation mutants, and found *DELLA* and *4CL7* had changes in amino acid. As shown in Fig. 7a, the base substitution causes the amino acid of *DELLA* to change from Gln to His in G2-G4. Similarly, the base substitution causes the amino acid of *4CL7* to change from Ala to Val in G1-G4 (Fig. 7b), and all non-synonymous were heterozygous mutations. Further domain analysis showed that changes of *DELLA* and *4CL7* amino acids did not cause domain changes in ‘Red Delicious’ and its four generation mutants (Fig. 7c, d). Thus, the above results showed that the non-synonymous SNP and InDel in spur-type and skin-color will consistence with the plant characteristic.

## Discussion

Color is an important index of fruit quality, which determines the appearance, nutritional value and economic benefit of fruit. The different colors of apple fruits were mainly caused by the difference of anthocyanin accumulation. Abundant apple germplasm resources and color mutants provide excellent materials for the study of plant anthocyanin metabolism [28]. In addition, the shape of the branches usually affects the yield and quality of the fruit. The fruit tree form with weak branch forming force and strong flower forming force is called spur-type, which is widely used in production because of its characteristics of high yield, good quality and easy management [29]. Bud sport often cause phenotypic changes, such as changes in the color, shape, ripening time and flavor of fruit, as well as in the size, shape and branching tendency of tree, while such phenotypic changes caused by somatic mutations may differ between different generations of the same plant [30–32]. In recent years, somatic mutations causing spur-type or skin-color alteration have been recognized in apple trees. For example, Xu et al. [33] found a mutant tree with blushed coloring pattern in fruit skin and identified as a bud sport of apple. Further research showed that color change of fruit skin is due to DNA methylation regulating anthocyanin biosynthesis by regulating *MdMYB1* expression, which affects apple color patterns. Furthermore, it has been proposed that miRNA159, miRNA167, miRNA396 and their potential targets and related phytohormone are important mechanisms regulating cell division and internode length in spur-type bud sport ‘Yanfu 6’ [34]. In this study, we found that G2, G3 and G4 have shorter internodes and smaller single leaf areas than G0 and G1 (Fig. 1a, b, d, e). Additionally, skin color and single fruit weight was increased with the increase of mutant generation (Fig. 1c, f). In particular, the main difference between G1 and G0 was an increase in the degree of the fruit skin color, but the coloration type still belonged to stripe red. Unlike G0 and G1, the mutants G2, G3 and G4 were spur type and all red (Fig. 2a, c). This is consistent with our previous findings that the pigmentation and anthocyanin content in fruit skin progressively increased from G0 to G4 [8]. These results preliminarily indicate that there are differences in multiple apparent morphologies caused by variation among ‘Red Delicious’ and its four generation mutants.

One of the goals of genomics is to identify the genetic loci responsible for variation in phenotypic traits. The completion of the apple genome sequence and recent advances in DNA sequencing technology allow for in-depth characterization of genetic variation present in the apple genome. The apple whole-genome resequencing and transcriptome have been widely used in research into response to more-branching, flowering and fruits coloring of apple. Xing et al. [35] re-sequenced two elite apple varieties ‘Nagafu No. 2’ and ‘Qinguan,’ and identified many genomic variations, which provided complex regulatory mechanisms involved in floral induction, flower bud formation and flowering characteristics. Besides, in another study, whole-genome resequencing and transcriptome were used to further investigate the molecular mechanisms of apple branching in wild-type apple (*M. spectabilis*) and its more-branching mutant [36]. Here, we applied whole-genome resequencing to analyze the variation of the apparent change in five generations. After comprehensive analysis of the sequencing results, it was determined that the sequencing quality was reasonable, and the data could detect the variation (Table 1; Supplementary Table 1). Meanwhile, whole-genome resequencing results showed that the number of SNPs, InDels and SVs were decreased in the four generation mutants relative to G0 (Table 1; Fig. 3a). This may be due to the genetic sequence of G1, G2, G3 and G4 being more similar to the apple reference genome GDDH13 v1.1. Sequence variation belongs to genetic variation and is usually manifested in SNPs, InDels, copy number variations (CNVs) and SVs, leading to large differences in the horticultural characters of plants [37, 38]. More importantly, the number of SVs found in the apple was much higher than in Arabidopsis [39], while fewer than in grape [38]. The major reason for this disparity is the differences of the level of genetic diversity within the population. Another reason is the retention of SVs > 20 bp in length, whereas only SVs > 50 bp in length were retained in this study.

SNP and InDel variants drive genome evolution [40]. In general, SNPs cause a higher frequency of genetic variation in the genome than InDels [41]. However, our study identified fewer InDels than SNPs, and similar results were found in apples and walnuts [22, 36]. Further results showed that chromosomes 2, 5, 11 and 15 had a large number of non-synonymous SNPs and InDels and SVs, while chromosomes 1 and 6 carried the least of which (Fig. 4). In general, regions with higher SNP frequency are shorter than regions with lower SNP frequency. The multiple regions with high-density or low-density SNPs and InDels distributed on the apple chromosomes will help to further localize and identify markers closely related to QTLs [42, 43]. In comparison with G0, the number of unique SNPs and InDels were significantly higher in G2 than G1, G3 and G4 (Supplementary Fig. 2). Besides, the numbers of differential non-synonymous SNPs and InDels, as well as the corresponding genes were also significantly higher in G2 than other three mutants (Supplementary Table 7). Therefore, the reason for the higher mutation frequency of G2 than G1, G3 and G4 was further investigated.

Phytohormone regulate various growth and development processes and play an important role in regulating plant height. GAs plays an important role in promoting internode and overall shoot elongation is mainly to regulated by cell differentiation, proliferation and expansion in plants [44]. Existing studies have shown that spur-type plants contain lower levels of GA compared to standard-type trees [45, 46]. Simultaneously, the alteration of one amino acid of GA receptor GID1 affects its interaction with DELLA protein and degradation of DELLA protein, resulting in peach dwarfing in peach trees [47]. In this study, the GA pathway gene *DELLA* (MD16G1023300) (Fig. 6c), which is closely related to the spur type of apple, was screened by KEGG enrichment analysis of the mutant DEGs. The structural integrity of DELLA protein itself is also essential for normal plant growth. The presence of a single SNP or InDel in a single gene sequence can alter plant type characteristics, and the deletion of 17 amino acids in the DELLA protein domain of Arabidopsis leads to the stunting phenotype [48]. In this study, DELLA protein is a inhibitory factor of GA signal, and gap-type mutations lead to a decrease of GA signal. Meanwhile, it was found that the expression level of *DELLA* was gradually reduced, and there was base substitution in the CDS region (Supplementary Table 10). It was speculated that the loss of partial function of this gene may be caused by base substitution in CDS region, which leads to shorter internode of branches. These results suggest that *DELLA* may be the gene that influences the spur-type traits of ‘Red Delicious’ and its four generation mutants.

Red is the most important feature of apple peel, a sign of fruit ripening, and an important factor in determining the quality of apple fruit. The red color of the skin is mainly due to the accumulation of anthocyanins. Anthocyanins are biosynthesized from phenylpropyl, which is the product of secondary metabolism of flavonoids and plays a decisive role in the color formation of apple peel [49]. In addition, color variation of fruit skin is mainly determined by the quantity and composition of anthocyanins [50, 51]. The genes involved in anthocyanin synthesis pathway are divided into structural and regulatory genes categories, and the expression of structural genes at the transcriptional level is activated by regulatory genes [52]. In our study, 16 genes related to skin-color were identified, of which five genes were up-regulated, mainly structural genes *4CL7*, *FLS/F3H*, *4CL2*, *PERP7* and *CHI*. *4CL* can catalyze the formation of cinnamyl coenzyme A from 4-coumaric acid, and plays an important role in regulating the biosynthesis of phenylpropanoids such as lignin, coumarin, flavonoids and chlorogenic acid [53]. The expression level of *4CL7* was significantly higher than that of other genes, suggesting that it plays a key role in regulating anthocyanin accumulation of ‘Red Delicious’ and its four generation mutants (Fig. 6d). The C/T base substitution occurred in CDS region of *4CL7* lead to changes in amino acids in G1-G4, and the expression level of this gene was increased (Supplementary Table 10). It was speculated that the base substitution made *4CL7* obtained function, and thereby changed skin color. The results indicated that the change of peel color of *4CL7* regulated ‘Red Delicious’ and its four generation mutants were related to base substitution in CDS region. In general, regulatory genes such as *MYB* are composed of transcription factors and are not directly involved in anthocyanin synthesis, but are involved in anthocyanin synthesis and accumulation by regulating the expression pattern and intensity of structural genes [54]. Whether *MYB* transcription factor regulates *4CL7*’s involvement in anthocyanin synthesis and accumulation needs to be further explored. Eventually, our results demonstrated that the identified non-synonymous SNPs and InDels can be used as a genomic resource to identify the genetic basis of trait variation.

## Conclusions

Bud sport breeding is an important way to select and breed high quality varieties in fruit cultivation. In this study, we investigated the differences in spur-type and skin-color between ‘Red Delicious’ and its four generation mutants by whole-genome resequencing and transcriptome. There were 4,491,880-4,682,649 SNPs, 710,292–752,869 InDels and 41,760 − 49,921 SVs contained in the individual samples. Chromosomes 2, 5, 11 and 15 were carried the most number of non-synonymous SNPs, InDels and SVs, while chromosomes 1 and 6 carried the least these. Moreover, 4,356 genes with structural variation were analyzed by KEGG functional annotation and identified 13 and 16 genes related to spur-type and skin-color from plant hormone signal transduction and phenylpropanoid biosynthesis pathways, respectively. Gene expression analysis showed that *DELLA* and *4CL7* were the main genes controlling the difference of spur-type and skin-color, respectively. These results provide the basis for apple trait variation and genetic improvement.

## Materials and methods

### Plant materials

‘Red Delicious’ (G0) and its four generation mutants including ‘Starking Red’ (G1), ‘Starkrimson’ (G2), ‘Campbell Redchief’ (G3), ‘Vallee spur’ (G4) were used as experimental materials. Red Delicious’ and ‘Starking Red’ grown in a demonstration base of Gansu Agricultural University at Wuwei in China. Other three mutants also grown in a demonstration base of Gansu Agricultural University at Tianshui in China. In September 2019, 15 apple trees aged 12 to 20 years in each generation were selected to collect samples, and the apple trees of the same generation were the same age. A replicate contains five fruit trees, and set three independent biological replicates. Each tree collected 10 leaves, 8–12 branches (a length of 40–70 cm) and 6 fruits. All samples were immediately brought back to the laboratory for further measurement of internode length, single leaf area and single fruit weight. The phloem and fruit skin samples were used to analyze the expression of genes related to spur-type and skin-color.

### Measurement of phenotypes indicators

The average internode length was estimated using the total stem length divided by the number of internodes. The Vernier caliper, YMJ-C leaf area measurement tool (Zhejiang Top Instrument Co., LTD., Hangzhou, Zhejiang, China), and electronic scale with 0.01 g sensitivity were used to measure the length of the shoot, the area of a single leaf, and the weight of single fruit, respectively.

### Whole-genome resequencing and quality control

Genomic DNA was extracted from leaves of the five generations from the pre-purified tachyzoites using the QIAamp® DNA Mini kit (QIAGEN, Germany), following the manufacturer’s protocol. The DNA samples were randomly sheared to 350 bp by the Covaris ultrasonic processor (Covaris, USA). Meanwhile, the DNA adaptors (Illumina, USA) with a ‘T’ base suspended at the 3’ end were ligated to DNA fragments with an ‘A’ base added to the end of the double-stranded broken DNA using T4 DNA polymerase. Resulting products were first separated on an agarose gel, and then extracted from the gel and purified. PCR amplification of adapter-modified DNA fragments was performed using Illumina paired-end PCR primers (Illumina, USA). The initial concentration of the library was measured using Qubit® 2.0 (Life technologies, USA). Next, the library concentration was further diluted to 1 ng/µL, and the insert size of the libraries were detected by Agilent Bioanalyzer 2100 (Agilent, USA). The libraries were sequenced on the Illumina HiSeq 2000 platform (Illumina, USA) and 150 bp paired-end reads by Novogene Bioinformatics Institute, Beijing, China. The effective concentration of the libraries was accurately determined by SYBR green qRT-PCR method. Original image data was converted to raw sequencing reads by base identification the base calling (Illumina pipeline CASAVA v1.8.2). Meanwhile, reads were filtered by set up quality control program: (1) containing Illumina library construction adapters; (2) containing more than 10% N bases; (3) More than 50% of reads with sequencing quality value below 5 were removed.

### Read mapping and unmap assembly

Sequencing reads were aligned to the apple reference genome GDDH13 v1.1 (https://iris.angers.inra.fr/gddh13/) [55] using BWA (parameter, mem -t 4 -k 32 -M) with default parameters [56]. Duplicate removal was performed using SAMtools (parameter, rmdup) [57] and PICARD (http://picard.sourceforge.net). Unmapreads were assembled with SOAPdenovo software, using a K-mer of 31, and different assembly contigs were obtained. The unmap sequence assembly results were evaluated comprehensively through the following conditions: (1) Contig length: the longer, the better; (2) GC content: the GC content of the assembled results should be close to the reference genome for near-source species; (3) Comparison of assembly results: the assembled reads as compared to the assembly results, the higher the coverage that the better the results; (4) Error rate detection: using contigs as the reference sequence, the detected homozygous SNP can be considered as error rate.

### Variant detection and annotation

The raw SNP and InDel sets were called by SAMtools with the parameters as ‘-q 1 -C 50 -m 2 -F 0.002 -d 1000’ [57]. SNPs with low support were removed. Then, filtering above sets with a mapping quality > 20 and a depth of variate position > 4. BreakDancer [58] and CNVnator [59] were used to detect SV and CNV, respectively. Five different types of SV sites can be predicted using Breakdancer (http://breakdancer.sourceforge.net/breakdancermax.html), including INS, DEL, INV, ITX and CTX. CNVnator can identify potential deletion numbers and duplication numbers by covering the depth of different reads on the genome. Variants were used by ANNOVAR for functional annotation, and genes and regions were annotated by known UCSC genes [60].

### RNA-Seq analysis

Transcriptome data was isolated from the results of our previous research [8]. ‘Red Delicious’ (G0) and its four generation mutants including ‘Starking red’ (G1), ‘Starkrimson’ (G2), ‘Campbell Redchief’ (G3) and ‘Vallee spur’ (G4) were selected as experiment materials. The 20–30 fruits of five generations were collected before the color break on 5 August (S1), at the color break on 25 August (S2), and during fruit maturity stages on 14 September (S3). Each treatment was set three independent biological replicates, and each replicate contained six fruits. The samples of S2 were used to RNA-Seq. A cDNA library was constructed by referencing the method of Mao et al. [61], and samples were sequenced on the Illumina HiSeq 2000 platform. Log_2_ FPKM ≥ 1.0 was used to identify DEGs, which were analysed by using GO and KEGG enrichment.

### Quantitative real-time PCR (qRT-PCR)

The total RNA of samples was extracted using plant RNA Extraction Reagent Kit (Real Times (Beijing) Biotechnology Co., Ltd.) and was reverse transcribed into cDNA by a Reverse Transcription Kit (Prime Script^RT^ reagent Kit, Perfect Real Time, TaKaRa). Meanwhile, gene-specific primers were designed on an online tool by Biotech Co. Ltd (Sangon, Shanghai), and primers details were shown in Supplementary Table 9. Then, we used the real time fluorescence PCR (LightCycler® 96 Real-Time PCR System, Roche, Switzerland) to detect the expression levels of genes. The reaction system was 20 µL, containing 6 µL ddH_2_O, 2 µL cDNA, 1 µL each of upstream and downstream primers and 10 µL SYBR Green (Accurate Biology), and the reaction procedure has been described in detail by Ren et al. [62]. The relative expression levels of genes were calculated using 2^*−∆∆CT*^ method [60], and the reference gene was *GADPH*.

### Data analysis

The data was analyzed by using IBM SPSS statistical 21. The data of average internode length, single leaf area and single fruit weight at each growth stage were evaluated by the ANOVA, and the results were presented as the mean values ± standard error (SE) of at least three independent experiments. Duncan’s multiple interval test (*P* < 0.05) was used to analyze the statistical differences between treatments.

### Electronic supplementary material

Below is the link to the electronic supplementary material.

Supplementary Material 1

## Data Availability

The datasets supporting the conclusions of this article are find with online web NCBI (https://www.ncbi.nlm.nih.gov/) PRJNA935103 and PRJNA935405.
