# Supplementary material for: Whole-genome resequencing and transcriptome analyses of four generation mutants to reveal spur-type and skin-color related genes in apple (Malus domestica Borkh. Cv. Red delicious)
Source: BMC Plant Biol. 2023 Nov 30;23:607. doi: 10.1186/s12870-023-04631-y (PMC10688089; doi:10.1186/s12870-023-04631-y)
Supplement: Supplementary file 1 — Supplementary Material 1 [file 12870_2023_4631_MOESM1_ESM.doc]

**
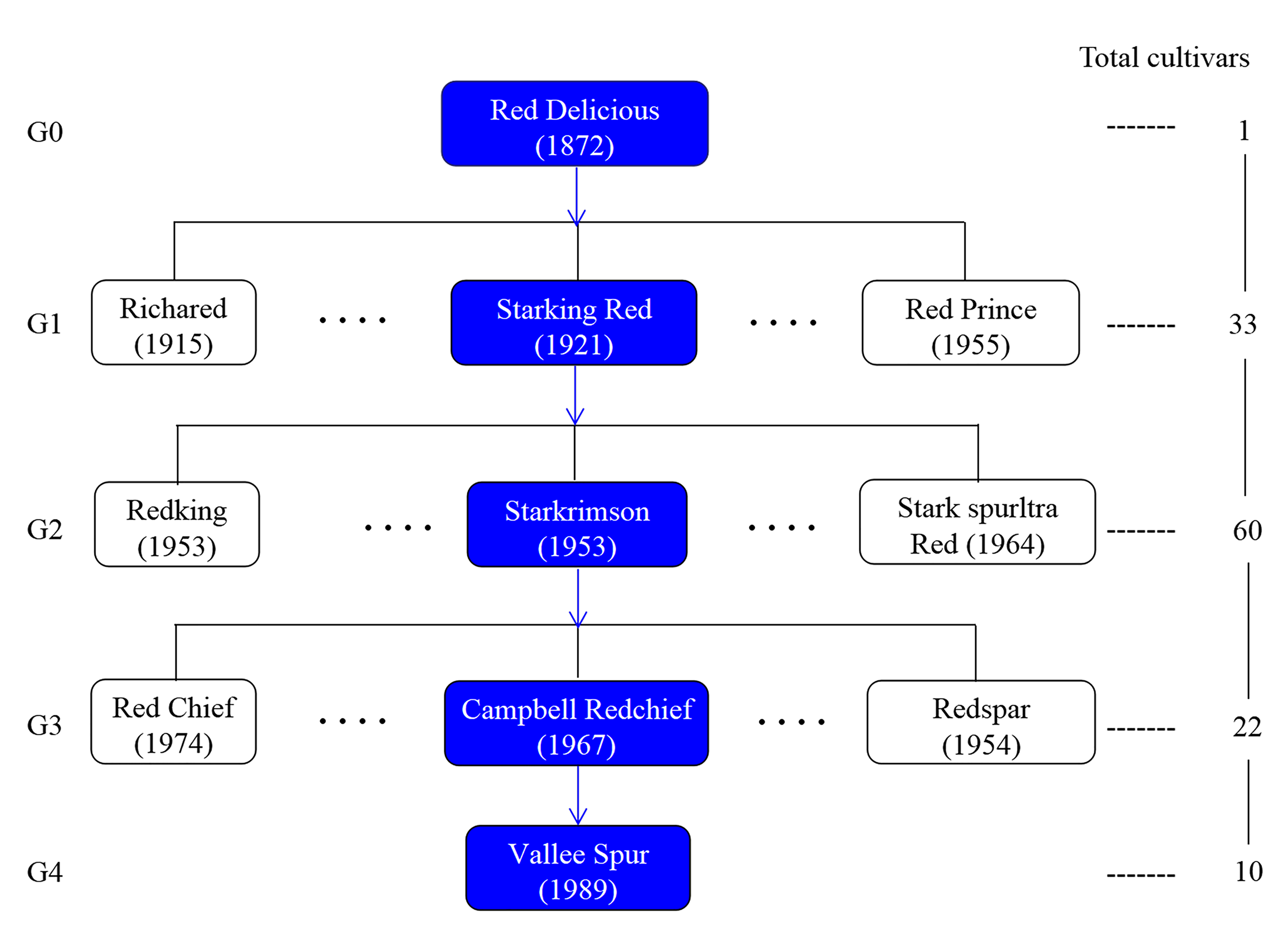
**

**Figure S1** Pedigree relationship of the sequenced apple samples. A rectangular box with blue color denotes show the samples from each generation were sequenced

**
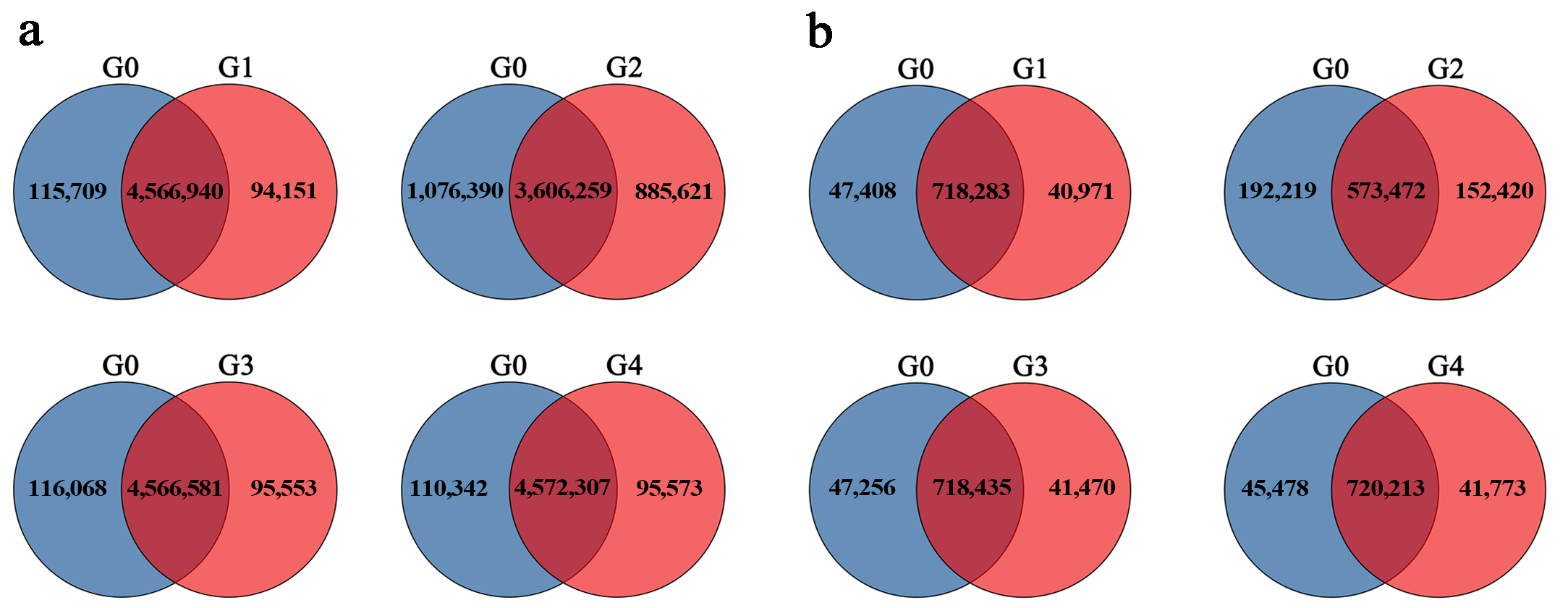
**

**Figure S2** Venn diagram of numbers of shared and specific SNPs (**a**) and InDels (**b**) between G0 (blue) and each mutant (red). G0, G1, G2, G3 and G4 represent ‘Red Delicious’, ‘Starking red’, ‘Starkrimson’, ‘Campbell Redchief’ and ‘Vallee spur’, respectively


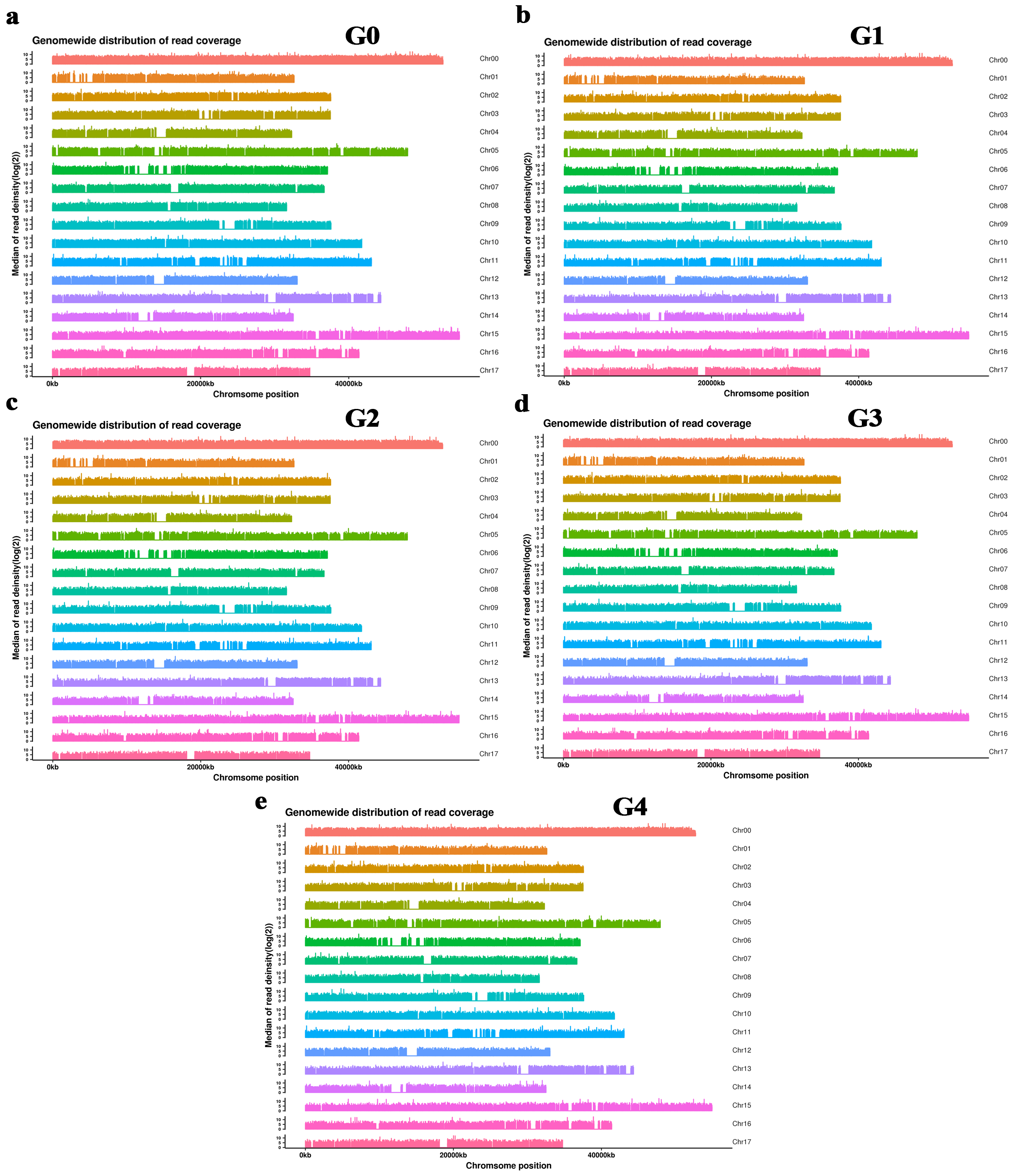


**Figure S3** The coverage depth distribution of individual points in the apple chr0 to chr17. The horizontal coordinate is the chromosome position, and the vertical coordinate is the value obtained by taking the log_2_ of the coverage depth at the corresponding position of the chromosome. G0, G1, G2, G3 and G4 represent ‘Red Delicious’, ‘Starking red’, ‘Starkrimson’, ‘Campbell Redchief’ and ‘Vallee spur’, respectively

**
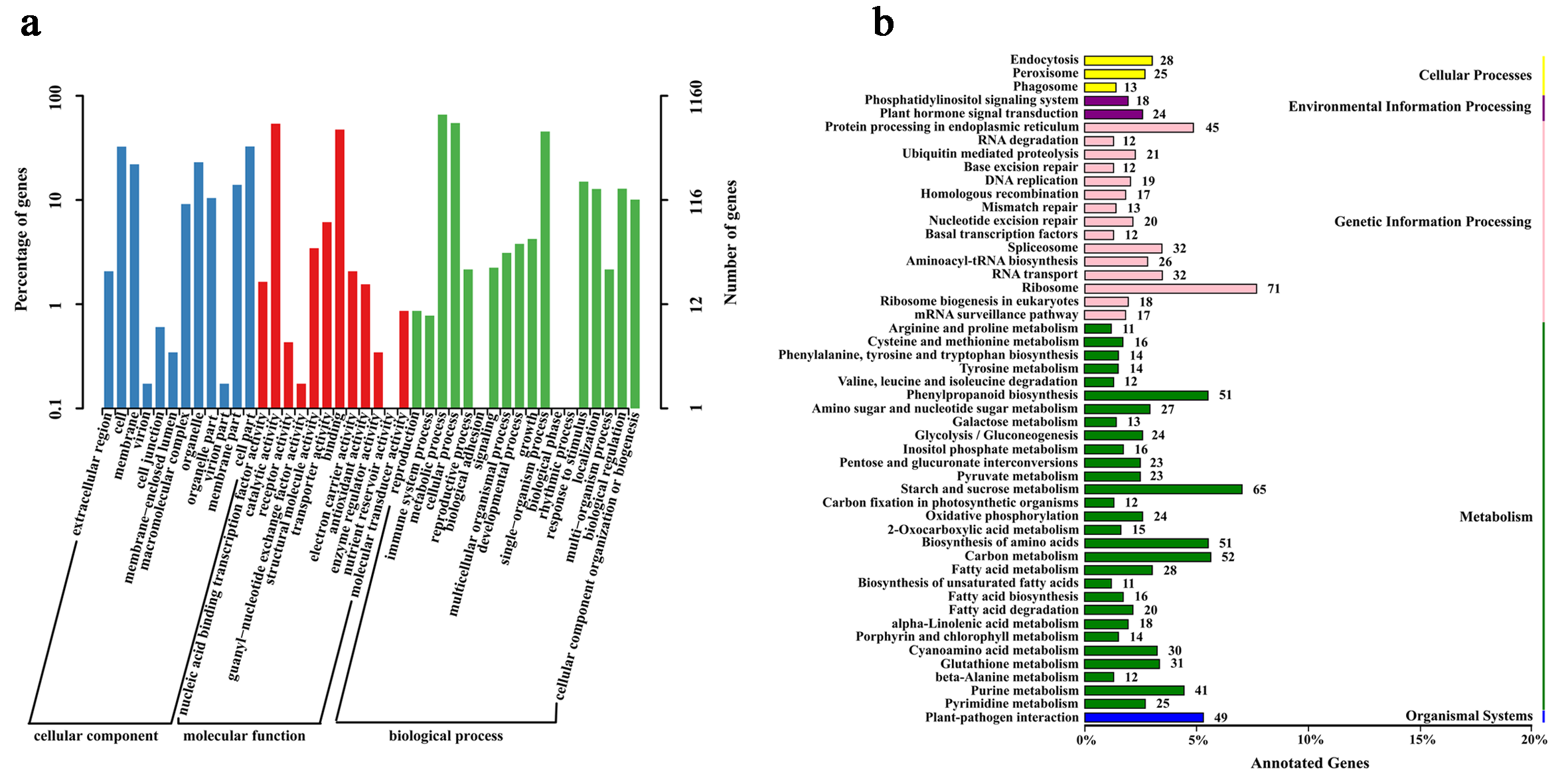
**

**Figure S4** GO (**a**) and KEGG (**b**) enrichment classification of variant genes in ‘Red Delicious’ (G0), ‘Starking red’ (G1), ‘Starking red’ (G2), ‘Campbell Redchief’ (G3) and ‘Vallee spur’ (G4). **a** The horizontal coordinate represents cellular component, molecular and biological process. The vertical coordinate represents the number and percentage of genes. **b** The horizontal coordinate represents the number and percentage of the annotated genes, and the vertical coordinate represents the pathway through which KEGG was enriched

**
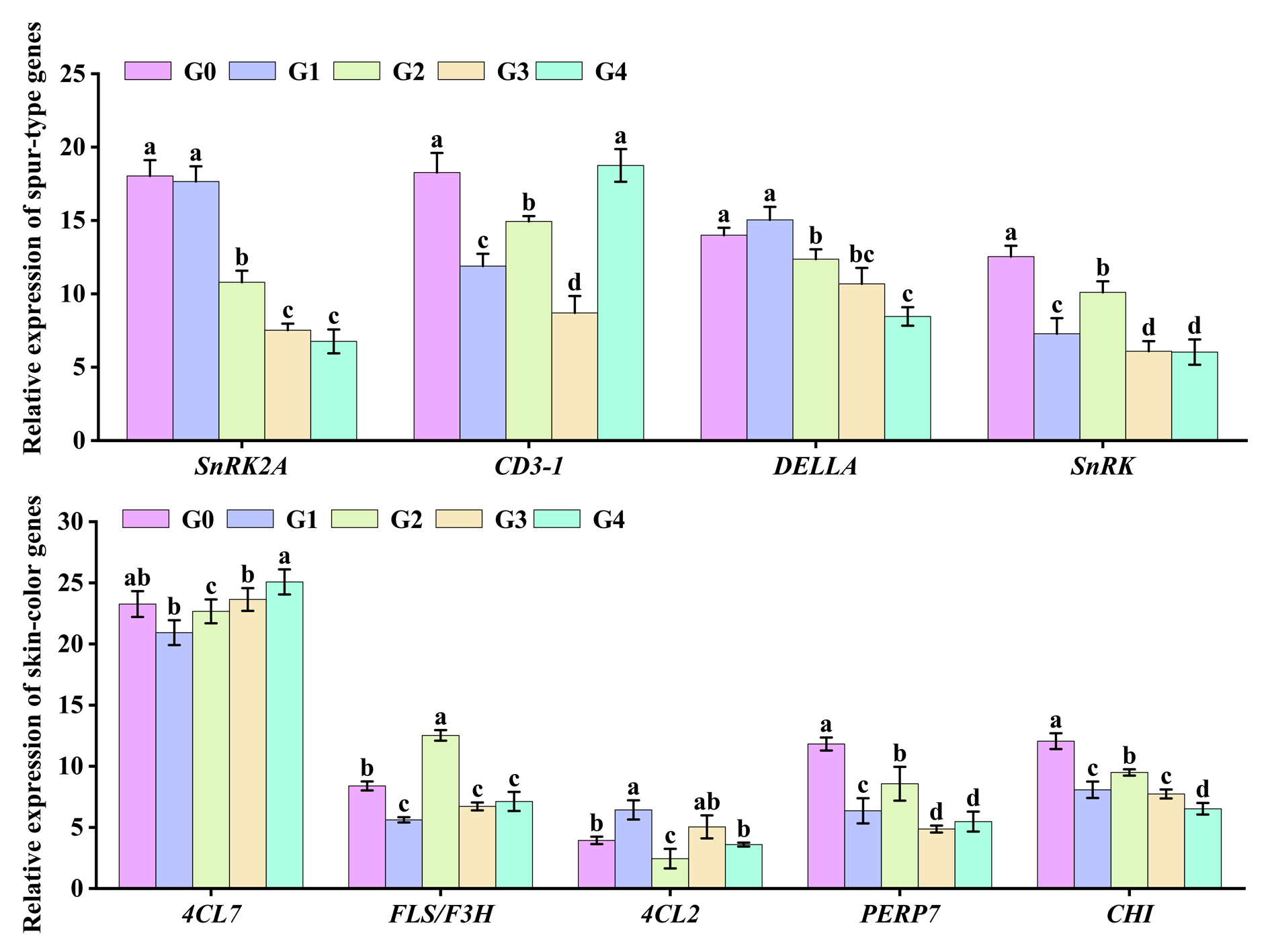
**

**Figure S5** qRT-PCR analysis of the variation DEGs related to spur-type and skin-color in ‘Red Delicious’ (G0), ‘Starking red’ (G1), ‘Starking red’ (G2), ‘Campbell Redchief’ (G3) and ‘Vallee spur’ (G4). Different small letters within the figures indicate significant differences at p < 0.05

**Supplementary Table 1** Summary of ‘Red Delicious’ and its four generation mutants sequencing

| **Sample** | **Raw Base (bp)** | **Clean Base (bp)** | **Effective Rate (%)** | **Error Rate (%)** | **Q20 (%)** | **Q30 (%)** | **GC Content (%)** |
| --- | --- | --- | --- | --- | --- | --- | --- |
| G0 | 53,026,443,300 | 52,954,836,900 | 99.86 | 0.03 | 96.76 | 92.27 | 38.18 |
| G1 | 33,518,576,400 | 33,469,927,500 | 99.85 | 0.03 | 97.23 | 93.19 | 38.23 |
| G2 | 35,192,771,700 | 35,147,940,600 | 99.87 | 0.03 | 97.07 | 92.84 | 38.22 |
| G3 | 32,927,064,600 | 32,880,054,900 | 99.86 | 0.03 | 96.94 | 92.49 | 38.27 |
| G4 | 34,979,318,100 | 34,929,156,900 | 99.86 | 0.03 | 96.93 | 92.50 | 38.29 |

# Note: G0, G1, G2, G3 and G4 represent ‘Red Delicious’, ‘Starking red’, ‘Starkrimson’, ‘Campbell Redchief’ and ‘Vallee spur’, respectively

# **Supplementary Table 2** Summary of ‘Red Delicious’ and its four generation mutants mapping

| **Sample** | **Total reads** | **Mapped reads** | **Average depth (×)** | **Coverage** **ratio 1× (%)** | **Coverage ratio 5× (%)** | **Coverage ratio 10× (%)** |
| --- | --- | --- | --- | --- | --- | --- |
| G0 | 353,032,246 | 342,088,246 (96.90%) | 76.00 | 96.71 | 95.22 | 94.09 |
| G1 | 223,132,850 | 217,331,395 (97.40%) | 46.00 | 96.39 | 94.50 | 92.95 |
| G2 | 234,319,604 | 228,859,957 (97.67%) | 48.00 | 96.46 | 94.64 | 93.17 |
| G3 | 219,200,366 | 214,202,597 (97.72%) | 46.00 | 96.39 | 94.49 | 92.96 |
| G4 | 232,861,046 | 227,272,380 (97.60%) | 49.00 | 96.41 | 94.57 | 93.11 |

# Note: G0, G1, G2, G3 and G4 represent ‘Red Delicious’, ‘Starking red’, ‘Starkrimson’, ‘Campbell Redchief’ and ‘Vallee spur’, respectively

**Supplementary Table 3** Summary of annotation for SNPs

| **Sample** | **Transitions (Ti)** | **Transversions (Tv)** | **Ti/Tv** | **Heterozygous rate (‰)** | **Total** |
| --- | --- | --- | --- | --- | --- |
|  |  |  |  |  |  |
| G0 | 3,196,395 | 1,486,254 | 2.15 | 2.444 | 4,682,649 |
| G1 | 3,181,288 | 1,479,806 | 2.14 | 2.436 | 4,661,094 |
| G2 | 3,062,229 | 1,429,651 | 2.14 | 2.342 | 4,491,880 |
| G3 | 3,182,443 | 1,479,691 | 2.15 | 2.436 | 4,662,134 |
| G4 | 3,186,396 | 1,481,484 | 2.15 | 2.439 | 4,667,880 |

# Note: G0, G1, G2, G3 and G4 represent ‘Red Delicious’, ‘Starking red’, ‘Starkrimson’, ‘Campbell Redchief’ and ‘Vallee spur’, respectively

**Supplementary Table 4** Summary of annotation for InDels

| **Sample** | **Insertion number** | **Deletion number** | **Heterozygous rate (‰)** | **Total** |
| --- | --- | --- | --- | --- |
|  |  |  |  |  |
| G0 | 364,581 | 401,110 | 0.352 | 765,691 |
| G1 | 360,510 | 398,744 | 0.358 | 759,254 |
| G2 | 344,710 | 381,182 | 0.368 | 725,892 |
| G3 | 360,734 | 399,171 | 0.358 | 759,905 |
| G4 | 361,877 | 400,109 | 0.357 | 761,986 |

# Note: G0, G1, G2, G3 and G4 represent ‘Red Delicious’, ‘Starking red’, ‘Starkrimson’, ‘Campbell Redchief’ and ‘Vallee spur’, respectively

**Supplementary Table 5** Summary of annotation for SVs

| **Sample** | **INS** | **DEL** | **INV** | **ITX** | **CTX** | **UN** | **Total** |
| --- | --- | --- | --- | --- | --- | --- | --- |
| G0 | 3,661 | 24,900 | 1,173 | 7,569 | 12,414 | 204 | 49,921 |
| G2 | 3,071 | 21,713 | 962 | 5,505 | 10,883 | 171 | 42,305 |
| G2 | 2,250 | 23,021 | 990 | 5,657 | 10,357 | 178 | 42,453 |
| G3 | 2,277 | 21,370 | 988 | 5,891 | 11,206 | 182 | 41,914 |
| G4 | 2,593 | 21,362 | 974 | 5,905 | 10,763 | 163 | 41,760 |

Note: G0, G1, G2, G3 and G4 represent ‘Red Delicious’, ‘Starking red’, ‘Starkrimson’, ‘Campbell Redchief’ and ‘Vallee spur’, respectively. INS, Insertions; Del, Deletion; INV, Inversions; ITX, Intra-chromosome transfers; CTX, Inter-chromosome transfers

# **Supplementary Table 6** Distribution of SVs and the type of variants per chromosome in ‘Red Delicious’ and its four generation mutants

| **Chromosome**  **genotype** | **SV number** | | | | |  | **CTX (%)** | | | | |  | **DEL (%)** | | | | |  | **INS (%)** | | | | |  | **INV (%)** | | | | |  | **ITX (%)** | | | | |
| --- | --- | --- | --- | --- | --- | --- | --- | --- | --- | --- | --- | --- | --- | --- | --- | --- | --- | --- | --- | --- | --- | --- | --- | --- | --- | --- | --- | --- | --- | --- | --- | --- | --- | --- | --- |
|  | **G0** | **G1** | **G2** | **G3** | **G4** |  | **G0** | **G1** | **G2** | **G3** | **G4** |  | **G0** | **G1** | **G2** | **G3** | **G4** |  | **G0** | **G1** | **G2** | **G3** | **G4** |  | **G0** | **G1** | **G2** | **G3** | **G4** |  | **G0** | **G1** | **G2** | **G3** | **G4** |
| chr0 | 2,206 | 1,874 | 1,872 | 1,829 | 1,795 |  | 32.09 | 33.14 | 33.60 | 34.45 | 32.87 |  | 43.02 | 45.30 | 48.44 | 44.61 | 46.52 |  | 7.61 | 7.52 | 5.02 | 5.36 | 5.74 |  | 1.04 | 1.23 | 1.60 | 1.31 | 1.62 |  | 15.54 | 12.70 | 11.97 | 13.89 | 12.70 |
| chr1 | 2,199 | 1,851 | 1,810 | 1,829 | 1,877 |  | 25.28 | 25.39 | 27.13 | 27.12 | 26.85 |  | 49.25 | 53.48 | 52.32 | 52.60 | 51.78 |  | 7.55 | 7.73 | 4.86 | 4.87 | 6.55 |  | 2.05 | 2.49 | 2.43 | 2.62 | 2.50 |  | 14.60 | 10.53 | 12.93 | 12.63 | 12.20 |
| chr2 | 3,077 | 2,556 | 2,856 | 2,566 | 2,586 |  | 16.31 | 17.64 | 14.43 | 18.67 | 17.48 |  | 54.40 | 54.58 | 60.08 | 54.36 | 54.64 |  | 6.86 | 7.51 | 4.38 | 4.83 | 6.34 |  | 3.93 | 3.83 | 3.82 | 3.70 | 4.02 |  | 17.81 | 15.85 | 16.88 | 18.04 | 16.94 |
| chr3 | 2,895 | 2,409 | 2,137 | 2,367 | 2,358 |  | 17.37 | 17.56 | 18.11 | 17.96 | 18.19 |  | 54.51 | 57.12 | 58.59 | 58.34 | 56.49 |  | 7.39 | 7.35 | 5.10 | 5.24 | 6.79 |  | 2.28 | 2.08 | 2.01 | 1.90 | 1.87 |  | 17.79 | 15.53 | 15.49 | 15.97 | 16.24 |
| chr4 | 1,899 | 1,639 | 1,889 | 1,657 | 1,613 |  | 12.11 | 13.91 | 11.49 | 13.76 | 12.77 |  | 62.93 | 63.33 | 66.70 | 63.25 | 63.30 |  | 7.16 | 7.38 | 6.19 | 6.28 | 6.88 |  | 1.84 | 2.32 | 1.91 | 2.17 | 2.23 |  | 15.48 | 12.93 | 13.45 | 14.12 | 14.45 |
| chr5 | 2,870 | 2,474 | 2,611 | 2,428 | 2,392 |  | 13.97 | 14.47 | 12.22 | 14.62 | 14.09 |  | 56.24 | 56.99 | 62.20 | 57.70 | 58.86 |  | 7.60 | 7.72 | 5.17 | 5.93 | 5.73 |  | 3.31 | 3.35 | 3.41 | 3.42 | 3.39 |  | 18.47 | 17.14 | 16.70 | 18.00 | 17.81 |
| chr6 | 1,735 | 1,457 | 1,532 | 1,478 | 1,502 |  | 7.26 | 7.41 | 6.92 | 8.59 | 7.52 |  | 68.93 | 67.33 | 69.91 | 67.73 | 66.51 |  | 7.49 | 7.48 | 5.61 | 5.35 | 7.12 |  | 1.67 | 1.72 | 2.09 | 1.69 | 1.80 |  | 17.69 | 15.85 | 15.01 | 16.44 | 16.78 |
| chr7 | 2,309 | 1,909 | 2,093 | 1,867 | 1,858 |  | 7.45 | 7.70 | 6.40 | 7.87 | 7.32 |  | 60.98 | 63.28 | 66.75 | 56.13 | 62.81 |  | 7.58 | 7.86 | 4.35 | 5.78 | 7.05 |  | 3.90 | 3.67 | 3.97 | 4.02 | 3.61 |  | 19.62 | 17.18 | 18.20 | 18.69 | 19.11 |
| chr8 | 1,670 | 1,330 | 1,231 | 1,371 | 1,379 |  | 0.00 | 0.00 | 0.00 | 0.00 | 0.00 |  | 65.03 | 70.60 | 70.59 | 76.59 | 68.38 |  | 9.52 | 6.62 | 7.15 | 6.13 | 7.47 |  | 4.55 | 4.81 | 4.39 | 5.03 | 5.15 |  | 20.66 | 17.67 | 17.71 | 19.84 | 18.78 |
| chr9 | 2,087 | 1,769 | 1,691 | 1,710 | 1,706 |  | 2.49 | 3.17 | 3.31 | 3.10 | 3.17 |  | 64.83 | 67.55 | 67.71 | 77.19 | 67.23 |  | 8.82 | 8.48 | 7.57 | 6.20 | 7.80 |  | 3.45 | 3.05 | 3.31 | 3.57 | 3.40 |  | 19.98 | 17.47 | 17.92 | 18.07 | 18.17 |
| chr10 | 4,079 | 3,401 | 3,428 | 3,352 | 3,374 |  | 36.16 | 36.99 | 35.88 | 37.68 | 37.11 |  | 39.99 | 40.84 | 44.02 | 36.52 | 40.57 |  | 7.55 | 7.23 | 5.37 | 5.67 | 6.22 |  | 2.33 | 2.26 | 2.16 | 2.03 | 1.90 |  | 13.31 | 12.20 | 12.08 | 12.65 | 13.60 |
| chr11 | 4,564 | 3,964 | 3,642 | 3,890 | 3,863 |  | 37.03 | 37.46 | 37.73 | 38.97 | 37.74 |  | 42.00 | 42.05 | 43.33 | 37.12 | 41.94 |  | 6.07 | 6.38 | 4.70 | 5.09 | 5.15 |  | 2.23 | 1.94 | 2.06 | 2.11 | 1.99 |  | 14.29 | 11.58 | 11.81 | 12.06 | 12.79 |
| chr12 | 3,123 | 2,675 | 2,467 | 2,691 | 2,648 |  | 33.56 | 32.49 | 35.47 | 35.45 | 33.61 |  | 45.31 | 46.84 | 46.66 | 42.33 | 46.71 |  | 5.60 | 5.94 | 4.99 | 4.57 | 5.06 |  | 1.67 | 1.35 | 1.22 | 1.30 | 1.36 |  | 13.51 | 12.82 | 11.27 | 12.71 | 12.95 |
| chr13 | 2,795 | 2,382 | 2,445 | 2,368 | 2,408 |  | 38.93 | 42.53 | 39.51 | 43.29 | 41.61 |  | 38.43 | 39.00 | 41.60 | 49.49 | 38.50 |  | 7.73 | 7.26 | 6.09 | 5.95 | 6.06 |  | 1.50 | 1.47 | 1.47 | 1.48 | 1.62 |  | 12.63 | 9.28 | 10.59 | 11.19 | 11.46 |
| chr14 | 2,468 | 2,110 | 2,184 | 2,065 | 2,107 |  | 30.51 | 30.24 | 28.94 | 31.23 | 31.51 |  | 38.82 | 50.14 | 54.03 | 47.26 | 48.88 |  | 7.78 | 8.20 | 5.17 | 5.91 | 6.50 |  | 1.42 | 1.52 | 1.83 | 1.89 | 1.76 |  | 11.63 | 9.43 | 9.48 | 11.43 | 11.06 |
| chr15 | 4,752 | 4,123 | 3,950 | 4,073 | 4,024 |  | 34.11 | 35.10 | 31.49 | 37.25 | 34.47 |  | 45.29 | 45.94 | 50.56 | 37.83 | 46.10 |  | 6.76 | 7.49 | 4.81 | 5.08 | 6.04 |  | 2.06 | 1.96 | 2.05 | 2.23 | 2.09 |  | 11.15 | 9.14 | 10.56 | 10.26 | 10.76 |
| chr16 | 2,871 | 2,466 | 2,397 | 2,461 | 2,423 |  | 27.83 | 28.75 | 26.87 | 28.57 | 28.35 |  | 51.86 | 53.33 | 56.57 | 27.02 | 53.61 |  | 6.44 | 5.47 | 4.63 | 5.00 | 4.95 |  | 1.25 | 1.34 | 1.13 | 1.34 | 1.49 |  | 12.57 | 10.71 | 10.55 | 11.21 | 11.39 |
| chr17 | 2,224 | 1,916 | 2,218 | 1,912 | 1,847 |  | 30.67 | 31.42 | 28.90 | 33.63 | 32.59 |  | 41.64 | 43.16 | 48.06 | 47.07 | 42.66 |  | 9.13 | 8.40 | 6.67 | 5.91 | 7.15 |  | 2.07 | 2.09 | 2.30 | 2.30 | 2.00 |  | 15.74 | 14.41 | 13.80 | 15.22 | 15.05 |

Note: G0, G1, G2, G3 and G4 represent ‘Red Delicious’, ‘Starking red’, ‘Starkrimson’, ‘Campbell Redchief’ and ‘Vallee spur’, respectively. CTX, Inter-chromosome transfers; Del, Deletion; INS, Insertions; INV, Inversions; ITX, Intra-chromosome transfers

# **Supplementary Table 7** Differential non-synonymous SNPs, InDels and its corresponding genes between ‘Red Delicious’ and its four generation mutants

|  |  | **SNPs** | |  | **InDels** | |
| --- | --- | --- | --- | --- | --- | --- |
|  |  | **Non-synonymous** | **Genes** |  | **Frameshift** | **Genes** |
| G0 versus G1 |  | 2,156 | 277 |  | 688 | 633 |
| G0 versus G2 |  | 27,976 | 6,233 |  | 4,680 | 3,783 |
| G0 versus G3 |  | 2,172 | 273 |  | 695 | 649 |
| G0 versus G4 |  | 2,117 | 275 |  | 679 | 632 |
| G1 versus G2 |  | 27,965 | 6,260 |  | 4,665 | 3,796 |
| G2 versus G3 |  | 27,980 | 6,244 |  | 4,667 | 3,830 |
| G3 versus G4 |  | 2,266 | 262 |  | 708 | 645 |

# Note: G0, G1, G2, G3 and G4 represent ‘Red Delicious’, ‘Starking red’, ‘Starkrimson’, ‘Campbell Redchief’ and ‘Vallee spur’, respectively

**Supplementary Table 8** Frameshift InDels information of 29 genes associated with spur-type and skin-color

| **#** | **Accession NO.** | **Chromosome** | **Annotated protein** | **G0** **versus G1** | **G0 versus G2** | **G0 versus G3** | **G0 versus G4** | **G1 versus G2** | **G2 versus G3** | **G3 versus G4** |
| --- | --- | --- | --- | --- | --- | --- | --- | --- | --- | --- |
| **Spur-type related genes** | | | | | | | | | | |
| 1 | MD05G1087300 | 5 | cyclin-D3-1 (*CD3-1*) |  | 8,531,956  frameshift insertion |  |  | 8,531,956  frameshift insertion | 8,531,956  frameshift insertion |  |
| 2 | MD09G1215800 | 9 | serine/threonine-protein kinase SAPK2-like (*SnRK2*) |  | 21,327,540  frameshift deletion |  |  | 21,327,540  frameshift deletion | 21,327,540  frameshift deletion |  |
| 3 | MD10G1134600 | 10 | serine/threonine-protein kinase (*SnRK*) |  | 21,662,983  frameshift insertion |  |  | 21,662,983  frameshift insertion | 21,662,983  frameshift insertion |  |
| 4 | MD14G1186200 | 14 | serine/threonine-protein kinase isoform X1 (*SnRKX1*) |  | 27,845,669  frameshift deletion |  |  | 27,845,669  frameshift deletion | 27,845,669  frameshift deletion |  |
| 5 | MD15G1279000 | 15 | serine/threonine-protein kinase SRK2I-like (*SnRK2I*) |  | 24,695,636  frameshift deletion |  |  | 24,695,636  frameshift deletion | 24,695,636  frameshift deletion |  |
| 6 | MD15G1330400 | 15 | transcription factor TGA4-like (*TGA4*) |  | 36,450,936  frameshift insertion |  |  | 36,450,936  frameshift insertion | 36,450,936  frameshift insertion |  |
| 7 | MD15G1413000 | 15 | BRASSINOSTEROID INSENSITIVE 1-associated receptor kinase 1-like (*OSI-BAK1*) |  | 51,258,524  frameshift insertion |  | 51,258,524  frameshift deletion | 51,258,524  frameshift insertion | 51,258,524  frameshift insertion | 51,258,524  frameshift deletion |
| 8 | MD15G1413300 | 15 | BRASSINOSTEROID INSENSITIVE 1-associated receptor kinase 3-like Family Protein (*OSI-BAK3*) |  | 51,293,039  frameshift insertion |  |  | 51,293,039  frameshift insertion | 51,293,039  frameshift insertion |  |
| 9 | MD15G1413600 | 15 | BRASSINOSTEROID INSENSITIVE 1-associated receptor kinase 6-like (*OSI-BAK6*) |  | 51,311,911  frameshift insertion |  |  | 51,311,911  frameshift insertion | 51,311,911  frameshift insertion |  |
| 10 | MD15G1428500 | 15 | serine/threonine-protein kinase SRK2A (*SnRK2A*) |  | 52,918,513  frameshift deletion |  |  | 52,918,513  frameshift deletion | 52,918,513  frameshift deletion |  |
| 11 | MD16G1023300 | 16 | DELLA protein (*DELLA*) |  | 1,699,443  frameshift insertion |  |  | 1,699,443  frameshift insertion | 1,699,443  frameshift insertion |  |
| 12 | MD16G1068400 | 16 | histidine kinase 3 isoform (*HK3*) |  | 4,792,743  frameshift insertion |  |  | 4,792,743  frameshift insertion | 4,792,743  frameshift insertion |  |
| 13 | MD16G1206500 | 16 | ARF domain class transcription factor (*ARF*) |  | 19,096,339  frameshift insertion |  |  | 19,096,339  frameshift insertion | 19,096,339  frameshift insertion |  |
| **Skin-color related genes** | | | | | | | | | | |
| 1 | MD01G1118300 | 1 | chalcone-flavonone isomerase (*CHI*) |  |  | 23,206,524  frameshift insertion |  |  | 23,206,524  frameshift insertion | 23,206,524  frameshift insertion |
| 2 | MD01G1162100 | 1 | peroxidase P7-like (*PERP7*) |  | 26,713,501  frameshift insertion |  |  | 26,713,501  frameshift insertion | 26,713,501  frameshift insertion |  |
| 3 | MD02G1259600 | 2 | beta-glucosidase BoGH3B-like isoform X1 (*BoGH3BX1*) |  | 31,289,778  frameshift deletion |  |  | 31,289,778  frameshift deletion | 31,289,778  frameshift deletion |  |
| 4 | MD03G1014200 | 3 | peroxidase A2-like (*PERA2*) | 31,289,778  frameshift deletion |  | 1,108,887  frameshift insertion |  | 31,289,778  frameshift deletion | 1,108,887  frameshift insertion | 1,108,887  frameshift insertion |
| 5 | MD05G1034100 | 5 | elicitor-activated gene 3-1 (*ELI3-1*) |  | 5,457,969  frameshift deletion | 5,457,969  frameshift deletion |  | 5,457,969  frameshift deletion | 5,457,969  frameshift deletion |  |
| 6 | MD06G1229100 | 6 | beta-glucosidase 44-likeb (*BGLU44*) |  | 35,894,970  frameshift insertion |  |  | 35,894,970  frameshift insertion | 35,894,970  frameshift insertion |  |
| 7 | MD07G1309000 | 7 | 4-coumarate-CoA ligase 2 (*4CL2*) |  | 36,439,182  frameshift deletion |  |  | 36,439,182  frameshift deletion | 36,439,182  frameshift deletion |  |
| 8 | MD08G1028600 | 8 | bifunctional dihydroflavonol 4-reductase/flavanone 4-reductase-like (*DFR*) |  | 2,083,420  frameshift insertion |  | 2,083,420  frameshift insertion | 2,083,420  frameshift insertion | 2,083,420  frameshift insertion | 2,083,420  frameshift insertion |
| 9 | MD09G1038600 | 9 | Peroxidase 63-like (*PER63*) |  | 2,352,630  frameshift deletion | 2,352,630  frameshift deletion | 2,352,630  frameshift deletion | 2,352,630  frameshift deletion |  |  |
| 10 | MD11G1100300 | 11 | beta-glucosidase 11-like (*BGLU11*) |  | 8,362,675  frameshift deletion |  |  | 8,362,675  frameshift deletion | 8,362,675  frameshift deletion |  |
| 11 | MD11G1145900 | 11 | 4-coumarate-CoA ligase-like 7 (*4CL7*) | 13,686,893  frameshift deletion | 13,686,893  frameshift deletion |  |  |  | 13,686,893  frameshift deletion |  |
| 12 | MD15G1022200 | 15 | peroxidase 9 (*PER9*) | 1,283,094  frameshift insertion |  |  |  | 1,283,094  frameshift insertion |  |  |
| 13 | MD15G1187300 | 15 | aldehyde dehydrogenase family 2 member C4-like (*ALDHC4*) |  |  | 14,792,993  frameshift deletion |  |  | 14,792,993  frameshift deletion | 14,792,993  frameshift deletion |
| 14 | MD15G1353800 | 15 | flavonol synthase/flavanone 3-hydroxylase-like (*FLS*/*F3H*) |  |  | 42,421,830  frameshift deletion | 42,421,830  frameshift deletion |  | 42,421,830  frameshift deletion | 42,421,830  frameshift deletion |
| 15 | MD17G1229400 | 17 | 4-coumarate-CoA ligase 1-like (*4CL1*) |  | 27,732,944  frameshift deletion |  |  | 27,732,944  frameshift deletion | 27,732,944  frameshift deletion |  |
| 16 | MD17G1265200 | 17 | peroxidase 52-like, partial (*PER52*) |  | 32,557,689  frameshift insertion |  |  | 32,557,689  frameshift insertion | 32,557,689  frameshift insertion |  |

# Note: G0, G1, G2, G3 and G4 represent ‘Red Delicious’, ‘Starking red’, ‘Starkrimson’, ‘Campbell Redchief’ and ‘Vallee spur’, respectively

# **Supplementary** **Table 9** The qRT-PCR primers used in the identification in ‘Red Delicious’ and its four generation mutants

| **Gene** | **Accession** | **Primer (5' → 3')** |
| --- | --- | --- |
| *SnRK* | MD10G1134600 | F: CGACCCACCACCCGAATCC |
|  |  | R: GCCTTCTCGCCGCTCTCAG |
| *DELLA* | MD16G1023300 | F: TGGCGGTCAACTCGGTCTTC |
|  |  | R: TCCACCACGGTCACAATCTCC |
| *CD3-1* | MD05G1087300 | F: CCAAGGAGGAGCAGGAACAGAC |
|  |  | R: CATCCACAGCACGGCATCAC |
| *SnRK2A* | MD15G1428500 | F: GTGCTCGGAAACATCCTTCT |
|  |  | R: TTCGCCACCCAAACATCATTCG |
| *4CL7* | MD11G1145900 | F: AGGGCTATGGTATGACAGAAACTTG |
|  |  | R: GAGGTTGAGGCTTCAGTGTATCTAC |
| *CHI* | MD01G1118300 | F: TCCACCGTCCGCCAAACC |
|  |  | R: GCCGTCTTACCCTTCCACTTAAC |
| *PERP7* | MD01G1162100 | F: ATTCCGCTCACGCATCTACAAC |
|  |  | R: GCTCCTGGTCTGAATGAAGAAGTC |
| *4CL2* | MD07G1309000 | F: GTCTGTCACTCGGCTATAACCAAC |
|  |  | R: CCTCATCGTCATCATCCACATAACC |
| *FLS/F3H* | MD15G1353800 | F: GAGCAGCCTGAGAACAGCAAAG |
|  |  | R: CCGCCATCCTCTTCACAACAAC |
|  |  | R: ACTTTTCAGCACCTTCGTGT |
| *GADPH* |  | F: TTCTCGTTGAGGGCTATTCCA |
|  |  | R: CCACAGACTTCATCGGTGACA |

**Supplementary Table 10** Statistical analysis of spur-type and skin-color genes non-synonymous SNP loci

| **#** | **Accession NO.** | **Chromosome** | **Reference chromosome** | **Gene** | **Full length** | **SNP non-synonymous location** | **Base substitutions** | | | | |
| --- | --- | --- | --- | --- | --- | --- | --- | --- | --- | --- | --- |
|  |  |  |  |  |  |  | **G0** | **G1** | **G2** | **G3** | **G4** |
| **Spur-type related genes** | | | | | | | | | | | |
| 1 | MD05G1087300 | 5 | NC_041793.1 | *CD3-1* | 47,952,461 | 18,669,199 | G/A | G/A | G/A | G/A | G/A |
|  |  |  |  |  |  | 18,669,539 | C/T | C/T | C/T | C/T | C/T |
|  |  |  |  |  |  | 18,669,551 | T/C | T/C | T/C | T/C | T/C |
|  |  |  |  |  |  | 18,669,595 | G/A | G/A | G/A | G/A | G/A |
|  |  |  |  |  |  | 18,669,651 | C/A | C/A | C/A | C/A | C/A |
|  |  |  |  |  |  | 18,670,268 | A/G | A/G | A/A | A/G | A/G |
| 2 | MD09G1215800 | 9 | NC_041797.1 | *SnRK2* | 37,604,908 | 21,327,634 | A/A | A/A | A/G | A/A | A/G |
|  |  |  |  |  |  | 21,331,513 | G/A | G/A | G/A | G/A | G/A |
| 3 | MD10G1134600 | 10 | NC_041798.1 | *SnRK* | 41,762,413 | 21,669,031 | G/C | G/G | G/C | G/C | G/C |
| 4 | MD14G1186200 | 14 | NC_041802.1 | *SnRKX1* | 32,513,452 | 27,848,153 | C/T | C/T | C/T | C/T | C/T |
|  |  |  |  |  |  | 27,854,223 | T/A | T/A | T/A | T/A | T/A |
|  |  |  |  |  |  | 27,854,436 | T/T | T/A | T/A | T/T | T/T |
|  |  |  |  |  |  | 27,855,140 | C/G | C/G | G/G | C/G | C/G |
|  |  |  |  |  |  | 27,855,200 | C/C | C/C | C/A | C/A | C/C |
|  |  |  |  |  |  | 27,855,340 | G/C | G/C | C/C | G/C | G/C |
| 5 | MD15G1279000 | 15 | NC_041803.1 | *SnRK2I* | 54,945,402 | 24,695,842 | A/C | A/C | A/A | A/A | A/C |
|  |  |  |  |  |  | 24,695,853 | T/C | T/C | T/T | T/T | T/C |
|  |  |  |  |  |  | 24,699,863 | C/T | C/T | C/T | C/T | C/C |
|  |  |  |  |  |  | 24,699,886 | A/G | A/G | A/G | A/G | A/G |
|  |  |  |  |  |  | 24,699,902 | G/G | G/G | G/A | G/G | G/G |
|  |  |  |  |  |  | 24,699,954 | C/A | C/A | C/A | C/A | C/A |
| 6 | MD15G1330400 | 15 | NC_041803.1 | *TGA4* | 54,945,402 | 36,456,907 | T/A | T/A | T/A | T/A | T/A |
|  |  |  |  |  |  | 36,457,667 | T/T | T/T | G/T | T/T | T/T |
|  |  |  |  |  |  | 36,457,707 | G/G | G/G | A/G | G/G | G/G |
|  |  |  |  |  |  | 36,458,954 | A/A | A/A | T/A | A/A | A/A |
| 7 | MD15G1413000 | 15 | NC_041803.1 | *OSI-BAK1* | 54,945,402 | 51,264,717 | T/C | T/C | C/C | T/C | T/C |
|  |  |  |  |  |  | 51,2647,26 | T/C | T/C | C/C | T/C | T/C |
|  |  |  |  |  |  | 51,264,834 | A/G | A/A | A/G | A/G | A/G |
|  |  |  |  |  |  | 51,264,861 | T/C | T/T | C/C | T/C | T/C |
|  |  |  |  |  |  | 51,264,932 | T/T | T/T | T/A | T/T | T/T |
|  |  |  |  |  |  | 51,264,967 | A/A | A/A | A/C | A/A | A/A |
|  |  |  |  |  |  | 51,265,010 | A/T | A/T | A/T | A/T | A/T |
|  |  |  |  |  |  | 51,265,062 | G/A | G/A | G/A | G/A | G/A |
|  |  |  |  |  |  | 51,265,102 | C/T | C/T | T/T | C/T | C/T |
|  |  |  |  |  |  | 51,265,135 | T/A | T/A | A/A | T/A | T/A |
|  |  |  |  |  |  | 51,265,557 | A/G | A/G | G/G | A/G | A/G |
|  |  |  |  |  |  | 51,266,943 | G/G | G/G | G/A | G/G | G/G |
| 8 | MD15G1413300 | 15 | NC_041803.1 | *OSI-BAK3* | 54,945,402 | 51,296,277 | C/C | C/C | C/G | C/C | C/C |
|  |  |  |  |  |  | 51,297,790 | A/A | A/A | A/T | A/A | A/A |
|  |  |  |  |  |  | 51,298,636 | G/G | G/G | G/A | G/G | G/G |
|  |  |  |  |  |  | 51,298,816 | A/A | A/A | A/G | A/A | A/A |
|  |  |  |  |  |  | 51,298,841 | C/C | C/C | C/G | C/C | C/C |
|  |  |  |  |  |  | 51,298,852 | T/T | T/T | T/G | T/T | T/T |
|  |  |  |  |  |  | 51,299,425 | A/A | A/A | A/G | A/A | A/A |
|  |  |  |  |  |  | 51,299,431 | G/G | G/G | G/C | G/G | G/C |
| 9 | MD15G1413600 | 15 | NC_041803.1 | *OSI-BAK6* | 54,945,402 | 51,312,190 | G/G | G/A | A/A | G/A | G/A |
| 10 | MD15G1428500 | 15 | NC_041803.1 | *SnRK2A* | 54,945,402 | 52,922,646 | T/G | T/G | T/G | T/G | T/G |
| 11 | MD16G1023300 | 16 | NC_041804.1 | *DELLA* | 41,389,449 | 1,699,270 | C/T | C/T | C/T | C/T | C/T |
|  |  |  |  |  |  | 1,699,278 | G/G | G/G | G/T | G/T | G/T |
|  |  |  |  |  |  | 1,699,288 | G/T | G/T | G/T | G/T | G/T |
|  |  |  |  |  |  | 1,699,350 | A/G | A/G | A/G | A/G | A/G |
|  |  |  |  |  |  | 1,699,376 | C/A | C/A | C/A | C/A | C/A |
|  |  |  |  |  |  | 1,699,382 | C/T | C/T | C/T | C/T | C/T |
|  |  |  |  |  |  | 1,699,390 | G/A | G/A | G/A | G/A | G/A |
|  |  |  |  |  |  | 1,699,421 | A/G | A/G | A/G | A/G | A/G |
| 12 | MD16G1068400 | 16 | NC_041804.1 | *HK3* | 41,389,449 | 4,792,575 | T/T | T/T | T/A | T/T | T/T |
|  |  |  |  |  |  | 4,792,607 | A/A | A/A | A/C | A/A | A/A |
|  |  |  |  |  |  | 4,792,650 | T/T | T/T | T/C | T/T | T/T |
|  |  |  |  |  |  | 4,792,668 | C/C | C/C | C/A | C/C | C/C |
|  |  |  |  |  |  | 4,792,694 | A/A | A/A | A/G | A/A | A/A |
|  |  |  |  |  |  | 4,792,716 | G/G | G/G | G/A | G/G | G/G |
|  |  |  |  |  |  | 4,792,809 | T/T | T/T | T/C | T/T | T/T |
|  |  |  |  |  |  | 4,792,818 | G/G | G/G | G/A | G/G | G/G |
|  |  |  |  |  |  | 4,792,827 | A/A | A/A | A/T | A/A | A/A |
|  |  |  |  |  |  | 4,792,842 | T/T | T/T | T/G | T/T | T/T |
|  |  |  |  |  |  | 4,795,799 | T/T | T/T | T/C | T/T | T/T |
| 13 | MD16G1206500 | 16 | NC_041804.1 | *ARF* | 41,389,449 | 19,102,241 | C/T | C/T | T/T | C/T | C/T |
| **Skin-color related genes** | | | | | | | | | | | |
| 1 | MD01G1118300 | 1 | NC_041789.1 | *CHI* | 32,625,452 | 23,211,911 | T/C | T/C | T/T | T/C | T/C |
| 2 | MD01G1162100 | 1 | NC_041789.1 | *PERP7* | 32,625,452 | 26,715,186 | T/A | T/A | T/T | T/A | T/A |
|  |  |  |  |  |  | 26,715,486 | A/C | A/C | A/A | A/C | A/C |
|  |  |  |  |  |  | 26,716,053 | C/C | C/C | C/A | C/C | C/C |
|  |  |  |  |  |  | 26,716,302 | C/T | C/T | C/C | C/T | C/T |
|  |  |  |  |  |  | 26,716,433 | A/C | A/C | A/C | A/C | A/C |
|  |  |  |  |  |  | 26,716,470 | C/C | C/C | C/A | C/A | C/C |
|  |  |  |  |  |  | 26,716,491 | G/G | G/G | G/A | G/G | G/G |
| 3 | MD02G1259600 | 2 | NC_041790.1 | *BoGH3BX1* | 37,577,729 | 31,290,410 | G/G | G/G | G/T | G/G | G/G |
|  |  |  |  |  |  | 31,290,486 | A/G | A/G | A/G | A/G | A/G |
|  |  |  |  |  |  | 31,290,492 | C/T | C/T | C/T | C/T | C/T |
|  |  |  |  |  |  | 31,290,603 | T/C | T/T | T/T | T/C | T/T |
|  |  |  |  |  |  | 31,291,342 | A/G | A/G | A/G | A/G | A/G |
|  |  |  |  |  |  | 31,291,433 | T/C | T/C | T/C | T/C | T/C |
|  |  |  |  |  |  | 31,291,843 | C/A | C/A | C/A | C/A | C/A |
|  |  |  |  |  |  | 31,291,888 | A/T | A/T | A/T | A/T | A/T |
|  |  |  |  |  |  | 31,292,101 | G/A | G/A | G/A | G/A | G/A |
| 4 | MD03G1014200 | 3 | NC_041791.1 | *PERA2* | 37,524,076 | 1,112,386 | C/C | C/C | C/G | C/C | C/C |
| 5 | MD05G1034100 | 5 | NC_041793.1 | *ELI3-1* | 47,952,461 | 5,464,781 | C/C | C/C | C/T | C/C | C/C |
|  |  |  |  |  |  | 5,465,736 | C/G | C/G | G/G | C/G | C/G |
| 6 | MD06G1229100 | 6 | NC_041794.1 | *BGLU44* | 37,137,259 | 35,902,384 | A/T | A/T | A/T | A/T | A/T |
| 7 | MD07G1309000 | 7 | NC_041795.1 | *4CL2* | 36,691,129 | 36,440,833 | T/G | T/T | T/G | T/G | T/G |
|  |  |  |  |  |  | 36,441,076 | G/A | G/A | G/A | G/A | G/A |
|  |  |  |  |  |  | 36,443,768 | A/C | A/C | C/C | A/C | A/C |
| 8 | MD08G1028600 | 8 | NC_041796.1 | *DFR* | 31,609,270 | 2,089,365 | A/T | A/T | A/T | A/T | A/T |
| 9 | MD09G1038600 | 9 | NC_041797.1 | *PER63* | 37,604,908 | 2,357,722 | T/T | T/T | T/G | T/T | T/T |
| 10 | MD11G1100300 | 11 | NC_041799.1 | *BGLU11* | 43,059,885 | 8,364,287 | C/T | C/T | C/T | C/T | T/T |
|  |  |  |  |  |  | 8,364,299 | T/C | T/C | T/C | T/C | T/T |
|  |  |  |  |  |  | 8,364,308 | C/T | C/T | C/T | C/T | C/T |
|  |  |  |  |  |  | 8,364,439 | T/T | T/T | T/C | T/T | T/T |
|  |  |  |  |  |  | 8,364,459 | T/C | T/C | T/C | T/C | T/C |
|  |  |  |  |  |  | 8,364,472 | A/T | A/T | A/A | A/T | A/T |
|  |  |  |  |  |  | 8,364,894 | T/T | T/T | T/A | T/T | T/T |
| 11 | MD11G1145900 | 11 | NC_041799.1 | *4CL7* | 43,059,885 | 13,694,480 | C/C | C/T | C/T | C/T | C/T |
|  |  |  |  |  |  | 13,694,780 | T/C | T/C | T/C | T/C | T/C |
| 12 | MD15G1022200 | 15 | NC_041803.1 | *PER9* | 54,945,402 | 1,283,355 | A/A | A/A | G/A | A/A | A/A |
|  |  |  |  |  |  | 1,283,449 | G/T | T/T | G/T | G/T | T/T |
|  |  |  |  |  |  | 1,283,468 | C/T | C/T | C/C | C/T | C/C |
|  |  |  |  |  |  | 1,283,581 | T/G | T/T | T/T | T/T | T/T |
|  |  |  |  |  |  | 1,284,084 | G/G | G/G | A/G | G/G | G/G |
| 13 | MD15G1187300 | 15 | NC_041803.1 | *ALDHC4* | 54,945,402 | 14,795,832 | G/A | G/A | G/A | G/A | G/A |
|  |  |  |  |  |  | 14,798,654 | C/C | C/C | A/C | C/C | C/C |
| 14 | MD15G1353800 | 15 | NC_041803.1 | *FLS*/*F3H* | 54,945,402 | 42,426,568 | G/T | G/T | G/G | G/T | G/T |
| 15 | MD17G1229400 | 17 | NC_041805.1 | *4CL1* | 34,748,701 | 27,738,351 | A/G | A/G | A/G | A/G | A/G |
|  |  |  |  |  |  | 27,738,597 | A/G | A/G | A/G | A/G | A/G |
|  |  |  |  |  |  | 27,738,704 | C/C | C/T | C/C | C/C | C/C |
|  |  |  |  |  |  | 27,738,729 | C/C | C/T | C/C | C/C | C/C |
|  |  |  |  |  |  | 27,739,142 | C/C | C/T | C/C | C/T | C/C |
|  |  |  |  |  |  | 27,739,148 | A/A | A/G | A/A | A/A | A/A |
|  |  |  |  |  |  | 27,740,906 | G/G | G/G | G/A | G/G | G/G |
| 16 | MD17G1265200 | 17 | NC_041805.1 | *PER52* | 34,748,701 | 32,558,622 | A/T | A/T | A/A | A/T | A/T |
|  |  |  |  |  |  | 32,559,720 | G/A | G/A | G/G | G/A | G/A |
|  |  |  |  |  |  | 32,559,742 | C/A | C/A | C/A | C/A | C/A |
|  |  |  |  |  |  | 32,560,196 | T/T | T/T | G/T | T/T | T/T |
|  |  |  |  |  |  | 32,560,391 | G/T | G/T | G/G | G/T | G/T |
|  |  |  |  |  |  | 32,560,438 | C/T | C/T | C/C | C/T | C/T |
|  |  |  |  |  |  | 32,560,518 | C/A | C/A | C/C | C/A | C/A |

# Note: G0, G1, G2, G3 and G4 represent ‘Red Delicious’, ‘Starking red’, ‘Starkrimson’, ‘Campbell Redchief’ and ‘Vallee spur’, respectively
